# Association of thyroid disease with risks of dementia and cognitive impairment: A meta-analysis and systematic review

**DOI:** 10.3389/fnagi.2023.1137584

**Published:** 2023-03-13

**Authors:** Li-Yun Ma, Bing Zhao, Ya-Nan Ou, Dan-Dan Zhang, Qiong-Yao Li, Lan Tan

**Affiliations:** Department of Neurology, Qingdao Municipal Hospital, Qingdao University, Qingdao, China

**Keywords:** dementia, cognitive impairment, thyroid disease, hyperthyroidism, hypothyroidism, meta-analysis

## Abstract

**Introduction:**

It is still uncertain whether the risk of dementia and cognitive impairment is related to thyroid disease. we carried out a meta-analysis and systematic review (PROSPERO: CRD42021290105) on the associations between thyroid disease and the risks of dementia and cognitive impairment.

**Methods:**

We searched PubMed, Embase, and Cochrane Library for studies published up to August 2022. The overall relative risk (RRs) and its 95% confidence interval (CIs) were calculated in the random-effects models. Subgroup analyses and meta-regression were conducted to explore the potential source of heterogeneity among studies. We tested and corrected for publication bias by funnel plot-based methods. The Newcastle-Ottawa Scale (NOS) or Agency for Healthcare Research and Quality (AHRQ) scale were used to evaluate the study quality of longitudinal studies and cross-sectional studies, respectively.

**Results:**

A total of 15 studies were included in our meta-analysis. Our meta-analysis showed that hyperthyroidism (RR = 1.14, 95% CI = 1.09–1.19) and subclinical hyperthyroidism (RR = 1.56, 95% CI = 1.26–1.93) might be associated with an elevated risk for dementia, while hypothyroidism (RR = 0.93, 95% CI = 0.80–1.08) and subclinical hypothyroidism (RR = 0.84, 95% CI = 0.70–1.01) did not affect the risk.

**Discussion:**

Hyperthyroidism and subclinical hyperthyroidism are predictors of dementia.

**Systematic review registration:**

PROSPERO, Identifier: CRD42021290105.

## 1. Introduction

Dementia is a progressive brain disease, which has brought heavy care and economic burdens to their families and society (Alzheimer's Association Report, 2021). It still has no effective disease-modifying treatment, and thus dementia prevention has become very important. And identifying modifiable risk factors and potentially reversible causes is crucial for the prevention of all-cause dementia (ACD). Alzheimer's disease (AD) is the most common type of dementia, followed by vascular dementia (VaD) (O'Brien and Thomas, 2015). Since thyroid function has been considered a potentially reversible cause of cognitive impairment, thyroid function analysis was recommended by the EFNS-ENS Guidelines as a routine assessment for the diagnosis of dementia (Knopman et al., 2001; Sorbi et al., 2012).

Hyperthyroidism (Hype) and hypothyroidism (Hypo) are very common thyroid diseases which exert potentially devastating effects on the elderly and they are characterized by abnormal thyroid-stimulating hormone (TSH) and free thyroid hormones (Taylor et al., 2018). The prevalence and incidence of thyroid diseases have been increasing globally (Taylor et al., 2018). There was evidence that thyroid diseases increased the risk of cardiovascular diseases and all-cause mortality (Manolis et al., 2020). However, it is still not clear whether thyroid diseases are risks factor for ACD. In addition, thyroid hormones are essential for neuronal development and cellular metabolism (Taylor et al., 2018). Hypo changed the morphology of some brain regions and subsequently altered the specific cognitive functions in a mouse model (Fernández-Lamo et al., 2009). Observational studies on the associations between Hype and the risk of cognitive disorders yielded inconsistent results. Some studies reported significantly increased the risks of dementia (Kalmijn et al., 2000; Vadiveloo et al., 2011; Folkestad et al., 2020), among people with Hype, while others showed no association (De Jongh et al., 2011; George et al., 2019; Folkestad et al., 2020). The same goes for observational studies on the links between Hypo with cognitive impairment (De Jongh et al., 2011; Forti et al., 2012) and ACD (Kalmijn et al., 2000; De Jong et al., 2006; Forti et al., 2012; Yeap et al., 2012; Aubert et al., 2017; George et al., 2019; Thvilum et al., 2021).

A previous meta-analysis reported a significant increase in the risk of cognitive impairment among people with subclinical hypothyroidism (SHypo) (Pasqualetti et al., 2015), while two other meta-analyses showed that SHypo was not associated with dementia risk (Akintola et al., 2015; Tang et al., 2021). The above three meta-analyses had not analyzed the associations between thyroid disease and dementia subtypes. Therefore, this meta-analysis is aimed to examine the association between thyroid disease and cognitive impairment, ACD (including dementia, AD and VaD). We performed a subgroup analysis according to the subtypes of thyroid disease (including Hype, Hypo, SHype, and SHype). We also conducted subgroup analyses stratified by population characteristics (region and age), follow-up years (≥5 years and < 5 years), and whether the studies were adjusted for cardiovascular and apolipoprotein E gene isoform 4 (*APOE*ε*4*) to deeply explore the effect of thyroid disease on cognitive impairment and dementia. Our study may have immense clinical implications for the prevention and treatment of dementia.

## 2. Methods

### 2.1. Protocol and registration

The systematic review was registered in advance with the international prospective register of systematic reviews (PROSPERO, registration number CRD42021290105) (Booth et al., 2012), following the Meta-analysis of Observational Studies in Epidemiology (MOOSE) guidelines (Stroup et al., 2000) and the Preferred Reporting Items for Systematic Reviews and Meta-Analyses (PRISMA) (Moher et al., 2009).

### 2.2. Search strategy

We systematically search in PubMed, EMBASE, and the Cochrane Library for relevant studies published from inception to May, 2022, using the following search strategy: ((((((((Thyroid Diseases) OR (Thyroiditis))) OR (Goiter)) OR (Hyperthyroidism)) OR (Hypothyroidism)) OR (Thyroid Dysgenesis)) OR (Thyroid Neoplasms) OR (thyrotoxicosis)) AND ((((Alzheimer^*^) OR (Dementia)) OR (Cognition)) OR (Cognitive)). There were no restrictions on the publication year. In addition, reference lists of previous review articles were hand-searched for additional relevant studies.

### 2.3. Study screening criteria and data extraction

Studies that fulfilled all of the following inclusion criteria were considered for inclusion: (1) they were published in English, (2) they used a cohort or cross-sectional study design to examine the association between thyroid disease and the risk of cognitive impairment or dementia, (3) they included participants aged > 18 years, and (4) they provided relative risks (RR), hazard ratios (HR), odds ratios (OR) with corresponding 95% confidence intervals (CI) or the raw data that can be utilized to calculate these numbers. Studies were excluded if they met any of the following criteria: (1) the risk estimate was not accessible, (2) only the abstract was available and (3) the article was a case report, an animal study, a letter to the editor or a comment. Additionally, if an article studied two different cohorts, both cohorts were included in our meta-analysis. Literature selection was conducted by two independent investigators (LYM and ZB), and any disagreement on study inclusion was discussed to reach a consensus. The following data were extracted from each studies: study characteristics, population characteristics, and result assessments. We would contact the corresponding authors if the above data was not available. A specific formula would be used to convert OR to RR when a study only reported OR (Xu et al., 2020). When we extracted the risk estimates in the article, the RR adjusted for the maximum number of covariates would be selected.

### 2.4. Evaluation of study quality

Two researchers independently evaluated the quality of enrolled cohort studies using the Newcastle-Ottawa Scale (Stang, 2010), including items in selection of study population, intergroup comparability, and determination of exposure/outcome with a total score of 9. Studies with scores of 0–3, 4–6, and 7–9 were assigned to low, medium, and higher-quality studies, respectively. The Agency for Healthcare Research and Quality (AHRQ) checklist, including 11 items, is recommended for quality assessment of cross-sectional studies (Zeng et al., 2015). Answers for each item are “yes,” “no,” and “unclear”. If you answer “yes” for each item, 1 point will be scored; otherwise, 0 point will be scored. Studies with a total score of 0–5, 6–7, 8–11 were defined as low, moderate and high quality, respectively (Zeng et al., 2015).

### 2.5. Statistical analysis

The overall risk ratios (RRs) and their 95% confidence intervals (CIs) from individual studies were pooled using a random-effects model (DerSimonian and Laird, 2015). Furthermore, forest plots were drawn to visually display the pooled results. The heterogeneity in the results across the studies was calculated using the I^2^ statistic which measured the percentage of the total variation across the included studies (low heterogeneity, I^2^ < 25%, moderate heterogeneity, 25% < I^2^ < 75%; high heterogeneity, I^2^ ≥ 75%) (Higgins and Thompson, 2002; Coory, 2010). For those with high heterogeneity or moderate heterogeneity, we adopted univariate regression analysis to find their sources of heterogeneity. Then, subgroup analyses was adopted to reduce the heterogeneity. For Longitudinal studies, subgroup analyses were performed by subtypes of dementia, types of thyroid disease (SHype and SHypo), as well as study characteristics (source of participants, follow-up duration and adjusted confounders) and participant characteristics (age, gender, and region). Publication bias was evaluated using Egger's test. The trim and fill methods were used to adjust for bias when statistically significant bias was found (Seagroatt and Stratton, 1998; Duval and Tweedie, 2000). Sensitivity analyses also were conducted for only the longitudinal studies to address the effect size.

All the statistical analyses were conducted using STATA (version 16.0, Stata Corporation, College Station, TX, USA) (Chaimani et al., 2014) R version 3.6.2 (https://www.R-project.org/). A two-sided *P* < 0.05 was considered statistically significant.

## 3. Results

### 3.1. Study search

**Figure 1:**
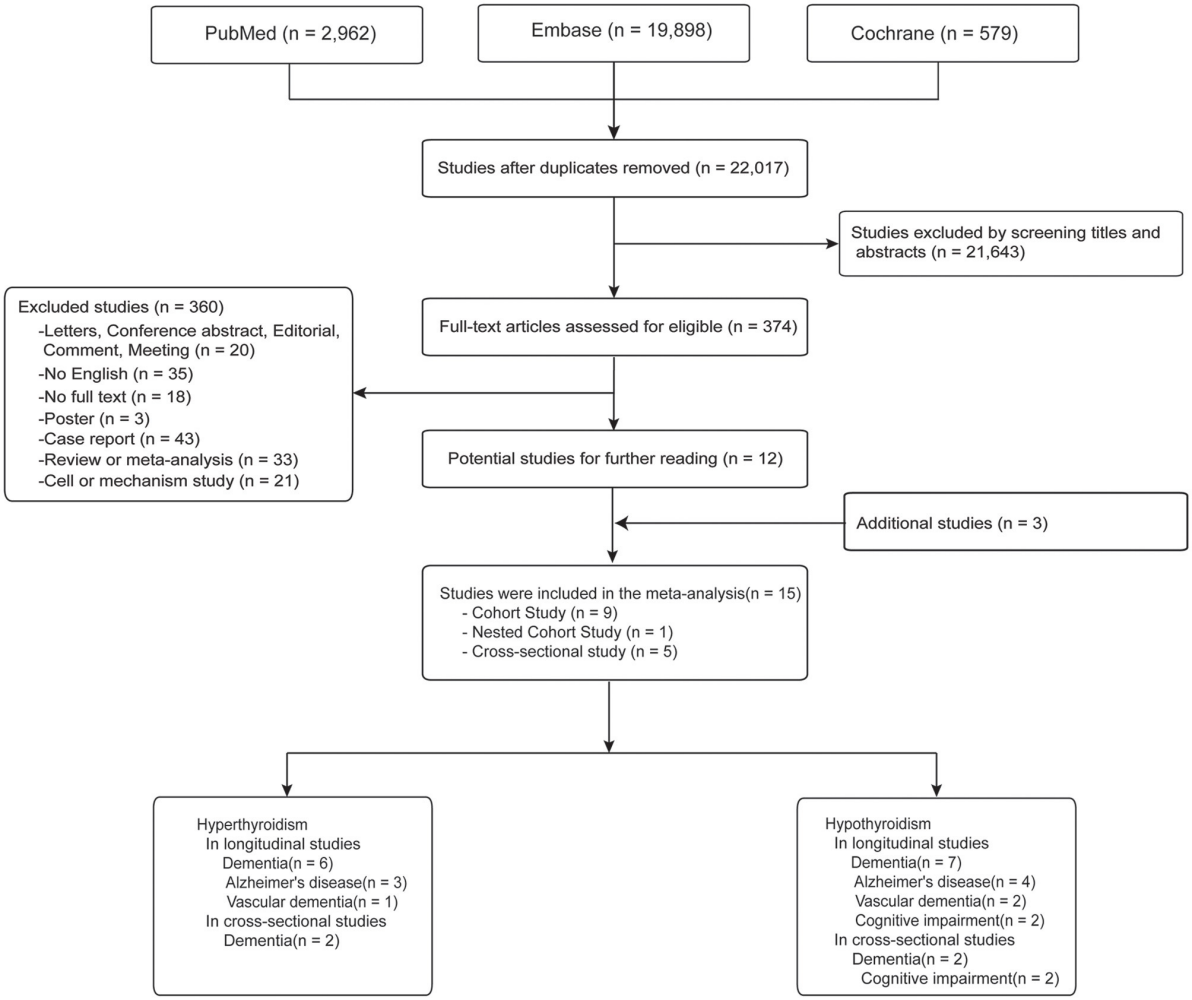
Flow diagram of literature selection for the meta-analysis.

Figure 1 presents the article selection process. There were 22,017 papers remained after deduplication. When we reviewed the titles and abstracts, 374 studies were included. After Detailed examining the full-text articles, a number of 12 studies met the inclusion criteria. After further integrating with additional three papers from the bibliography, a final total of 15 studies (ten longitudinal studies and five cross-sectional studies) were finally included.

### 3.2. Characteristics of studies

**Figure 2:**
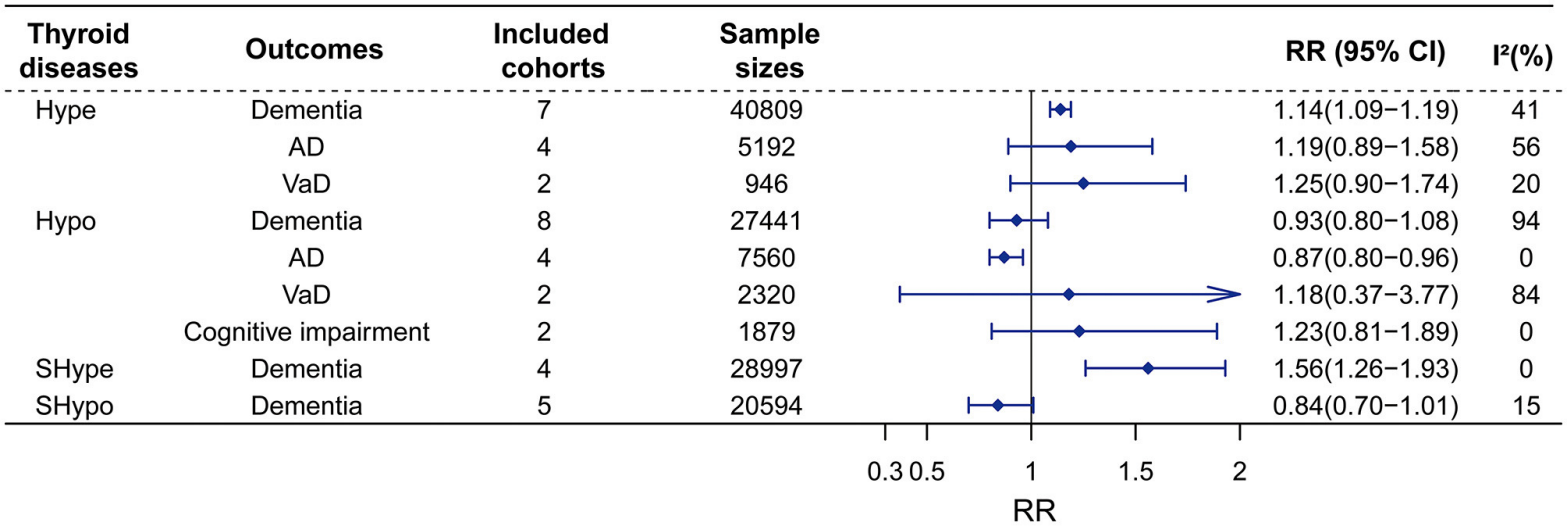
Meta-analysis of associations between thyroid disease and cognitive outcomes in the longitudinal study. Hype would increase the risks of dementia, whereas would not affect the risk of AD, or VaD. Besides, Hypo was no associated with dementia, VaD, and cognitive impairment, but was found to reduce the risk of AD. Furthermore, SHype would advance the risk of dementia, but SHypo was found to reduce the risk of dementia. RR, relative risk; CI, confidence interval; AD, Alzheimer's disease; VaD, vascular dementia; Hype, hyperthyroidism; Hypo, hypothyroidism; SHype, subclinical hyperthyroidism; SHypo, subclinical hypothyroidism.

The detailed characteristics of studies included in the meta-analysis are shown in Table 1. Ten longitudinal studies (Kalmijn et al., 2000; De Jong et al., 2006; De Jongh et al., 2011; Vadiveloo et al., 2011; Forti et al., 2012; Yeap et al., 2012; Aubert et al., 2017; George et al., 2019; Folkestad et al., 2020; Thvilum et al., 2021) published between 2000 and 2021 with 298,522 patients (minimum sample size *n* = 660; maximum sample size *n* = 233,844). The participants had a mean age of 66.81 years, with a female proportion of 58.71%. Among these studies, six cohort studies reported the association of Hype with dementia (Figure 2) (Kalmijn et al., 2000; De Jong et al., 2006; Vadiveloo et al., 2011; Aubert et al., 2017; George et al., 2019; Folkestad et al., 2020), AD (Kalmijn et al., 2000; De Jong et al., 2006; Folkestad et al., 2020) or VaD (Folkestad et al., 2020); seven cohort studies reported the association of Hypo with dementia (Figure 2) (Kalmijn et al., 2000; De Jong et al., 2006; Forti et al., 2012; Yeap et al., 2012; Aubert et al., 2017; George et al., 2019; Thvilum et al., 2021), AD (Kalmijn et al., 2000; De Jong et al., 2006; Forti et al., 2012; Thvilum et al., 2021), VaD (Forti et al., 2012; Thvilum et al., 2021) or cognitive impairment (De Jongh et al., 2011; Forti et al., 2012).

**Table 1:** Characteristics of studies included in the meta-analysis.

**References; cohort name; region; source; study**	**Age [mean (SD)], (years); female (%)**	**Hormone or thyroid medication users excluded**	**Dementia at baseline excluded**	**Mean follow-up (SD), years, Follow-up rate**	**Thyroid diseases**	**Outcome**	**Diagnosis criteria**	**Adjusted factors**
Kalmijn et al. (2000); TRS; Netherlands; population; cohort	68.8 (7.5); 72	Yes	Yes	9;0.85	SHype; SHypo	Dementia; AD	DSM-III-R (dementia), NINCDS/ADRDA (AD)	Age and gender
De Jong et al. (2006); TRSS; Netherland; population; cohort	72.3 (7.4); 51	Yes	Yes	5.5;UC	Hype; Hypo	Dementia; AD	DSM-III-R(dementia), NINCDS/ADRDA(AD)	Age and gender
Forti et al. (2012); CSBA; Italy; population; cohort	73.3 (6.0); 53	NO	Yes	3.8 (0.7); 0.8	Hypo	Dementia; AD; VaD; MCI	DSM-IV (dementia); NINCDS/ADRDA (AD; NINDS/AIREN (VaD), I-D-C (MCI)	Age, gender and education, r serum cholesterol, Geriatric Depression Scale, BMI, hypertension, diabetes, history of cardiovascular disease and plasma total homocysteine
Yeap et al. (2012); HIMS; Australia; population; cohort	78.3; 0	Yes	Yes	5.9;0.98	SHypo	Dementia	ICD-9 and ICD-10	Crude
De Jongh et al. (2011)^※^; LASA; Netherland; population; cohort	75.5;50.5	Yes	NA	UC	SHype; SHypo	Cognitive impairment	MMSE	Age, gender, alcohol use, smoking status, educational level, number of chronic diseases, and physical activity.
Aubert et al. (2017); Health ABC Study; U.S.A; population; cohort	75.1 (2.8); 52	Yes	Yes	9;0.85	SHype; SHypo	Dementia	Dementia was defined by any of the following criteria※※	Age, sex, race, education level and 3MS at baseline. There were 7 missing values for education level, all in participants with euthyroidism
George et al. (2019); ARIC; U.S.A; community; cohort	57 (5.7);56	NO	Yes	21.9;0.91	SHype; SHypo; Hype; CHypo; Hype; Hypo	Dementia	ICD-9	Age, sex, race-center, APOE ε4, income and education. BMI, smoking status, hypertension, diabetes, drinking status, HDL cholesterol and total cholesterol, prevalent CVD and baseline thyroid medication use.
Folkestad et al. (2020); DNPR, OPENTHYRO; Denmark; Registry; nested case-control	60, 62.5; NC, 77	Yes	Yes	7.2,7.3; near100%	Hype; Hype,	Dementia; AD; VaD	ICD-10	Crude
Vadiveloo et al. (2011); TEARS;U.K; population; cohort	66.5 (15.9), 77	Yes	NC	5.6	SHype	Dementia	ICD-9;ICD-10	History of dementia, history of psychiatric, and age × gender interaction
Thvilum et al. (2021); DNPR, OPENTHYRO; Denmark; population; cohort	56; 83 56.4; 56	Yes	Yes	6.2;7.2	Hypo	Dementia; AD	ICD10 (dementia), prescription of medicine for dementia.	Charlson Comorbidity Index; crude
Cárdenas-Ibarra et al. (2008); NA; Mexica; Hospital; Cross-sectional	74.8 (8.0); 69	NC	NC	NA	Hype; Hypo	Cognitive impairment	MMSE	Age, sex, race, smoking history, and educational attainment
Park et al. (2010); KLoSHA; Korean; populatio; Cross-sectional	65.53 (11.87); 43	Yes	Yes	NA	SHypo	AD; cognitive impairment	Consortium to Establish a Registry for Alzheimer's Disease (CERAD), MMSE	Age, sex and duration of education
Parsaik et al. (2014); NA; U.S.A; population; Cross-sectional	80.34;48.6	Yes	Yes	NA	Hypo; SHypo; CHypo	Mild cognitive impairment	CDR, FAQ, DSM-IV	Age, gender, education, education years, sex, ApoE ε 4, depression, diabetes, hypertension, stroke, BMI and coronary artery disease)
Benseñor et al. (2010); SPAH; Brazil; population; Cross-sectional	75.63;60.5	Yes	NC	NA	SHype;	Dementia, AD; VaD	DSM-IV	Age
Bajaj et al. (2014); NA; India; Hospital; Cross-sectional	75; 57.8	Yes	Yes	NA	SHypo	Cognitive impairment	MMSE	Age, sex and education

Health ABC Study: the Health, Aging and Body Composition Study; ARIC, Atherosclerosis Risk in Communities Neurocognitive Study; TRS, the Rotterdam Study; TRSS, the Rotterdam Scan Study; TEARS, The Thyroid Epidemiology, Audit, and Research Study; CSBA, the Conselice Study of Brain Aging; DNPR, Danish National Patient Registry; OPENTHYRO, Odense Patient data Explorative Network Thyroid Status and Register Outcomes; TEARS, The Thyroid Epidemiology, Audit, and Research Study; LASA, the Longitudinal Aging Study Amsterdam; SPAH, the Sáo Paulo Aging and Health Study; HIMS, Health in Men Study; AD, Alzheimer's disease; VaD, Vascular dementia; MCI, Mild cognitive impairment; Hype: hyperthyroidism; Hypo, hypothyroidism; SHype, Subclinical hyperthyroidism; SHypo, Subclinical hypothyroidism; CHype, clinical hyperthyroidism; CHypo, clinical hypothyroidism; NA, Not Applicable; UC, unclear.

※: In Baseline data. UC, unclear.

※※: Dementia was defined by any of the following criteria: (i) ≧ 1.5 SD race- stratified 3MS decline from Year 1 visit to last available examination; (ii) dementia as primary or secondary diagnosis of admission on hospital records with 3MS ≦ 90 points at Year 3 or either follow- up; (iii) prescription of dementia drug recorded on the yearly drug inventory.

Five cross-sectional studies (Cárdenas-Ibarra et al., 2008; Ceresini et al., 2009; Benseñor et al., 2010; Park et al., 2010; Bajaj et al., 2014; Parsaik et al., 2014) published between 2009 and 2014 with a total of 4,248 individuals were included (minimum *n* = 44; maximum *n* = 1,904). The participants had a mean age of 76.49 years with a female proportion of 55.90%. Among these studies, two studies reported the association of Hype with dementia (Cárdenas-Ibarra et al., 2008; Benseñor et al., 2010), and two studies reported the association of Hypo with cognitive impairment (Bajaj et al., 2014; Parsaik et al., 2014) or dementia (Cárdenas-Ibarra et al., 2008; Park et al., 2010). The Newcastle-Ottawa Quality Assessment scale and the AHRQ scale were used to evaluate the study quality of longitudinal studies and cross-sectional studies, respectively. All the included studies were identified as being of moderate-to-high quality (Supplementary material 1).

#### 3.2.1. Longitudinal studies (including cohort studies and nested case-control studies)

##### 3.2.1.1. Hype and dementia

**Figure 3:**
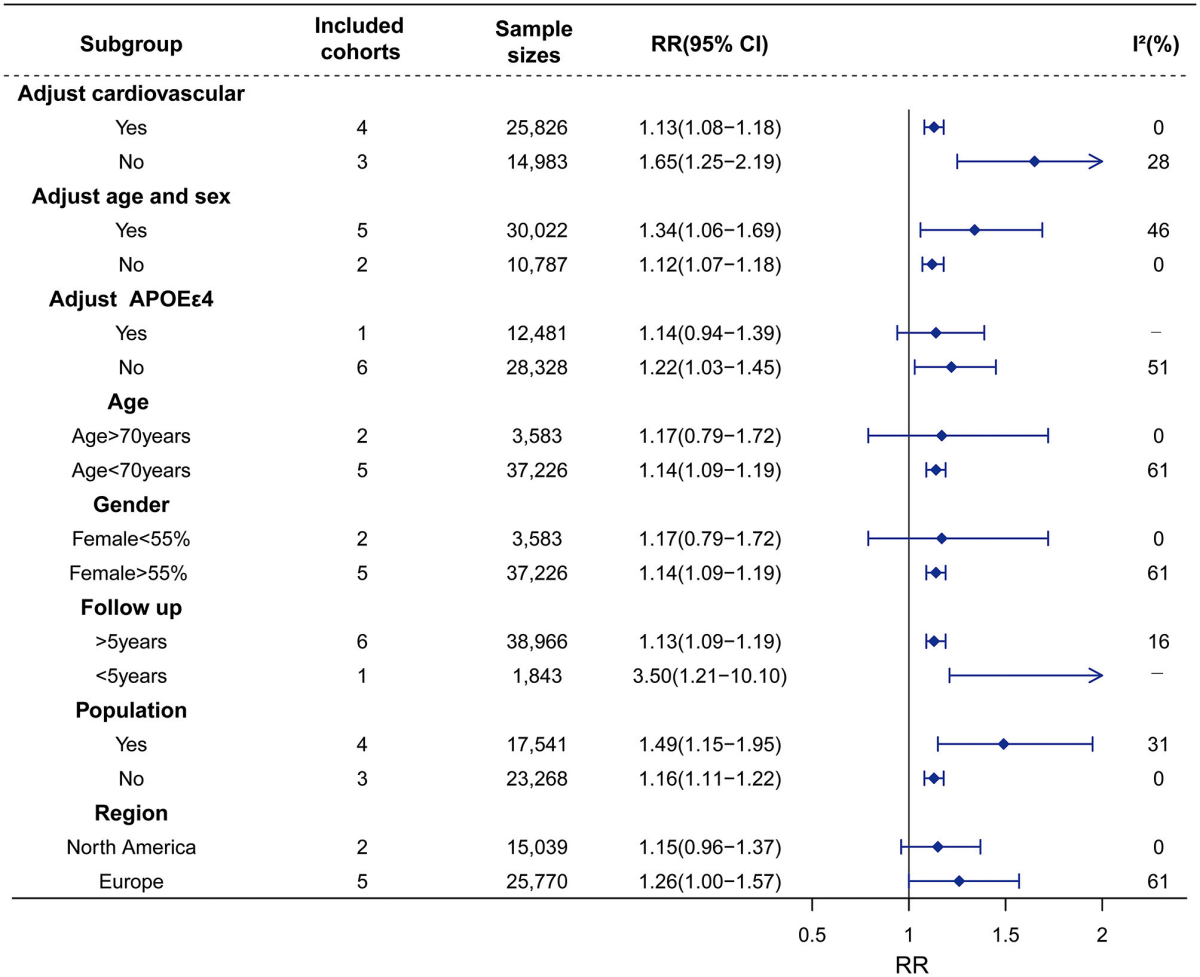
Association of Hype with risk of dementia in the longitudinal study. Subgroup analyses indicated that the significance of the primary result was not altered by adjustment factors (vascular factor, age, sex), follow-up, and source of cohorts. However, the pooled results tend to be non–significant in adjusting factor of APOEε4, age > 70 years, female < 55%, and region in North America. The heterogeneity was reduced in the sub-analysis of adjustment factors (vascular factor), and source of cohorts. RR, relative risk; CI, confidence interval; AD, Alzheimer's disease; Hype, hyperthyroidism; *APOE*ε*4*, apolipoprotein E gene isoform 4.

Sex studies on Hype with 40,809 participants were included. The results of our meta-analyses showed that Hype significantly increased the risk of dementia (RR = 1.14, 95% CI = 1.09–1.19, I^2^ = 41%) rather than AD (RR = 1.19, 95% CI = 0.89–1.58, I^2^ = 56%) or VaD (RR = 1.25, 95% CI = 0.90–1.74, I^2^ = 20%) (Figure 2; Supplementary material 2). Meta-regression revealed that no factors could explain the source of heterogeneity (Supplementary material 3). Sensitivity analysis showed that the robust results were not influenced by any single study (Supplementary material 4). Subgroup analyses indicated that the sources of heterogeneity in the primary results were adjustment for cardiovascular factors and the source of population rather than adjustments for age, sex and APOEε4, follow-up duration (>5 years vs. < 5 years), age (>70 years vs. < 70 years), female proportion (>55% vs. < 55%) or region (Figure 3; Supplementary material 5). Publication bias in the studies on the association of Hype and dementia was evaluated by funnel plots and Egger's test. Egger's test suggested no statistically significant publication bias (*p* = 0.299). A symmetric funnel plot (Supplementary material 6) also indicated no evidence of publication bias.

##### 3.2.1.2. Hypo and dementia

**Figure 4:**
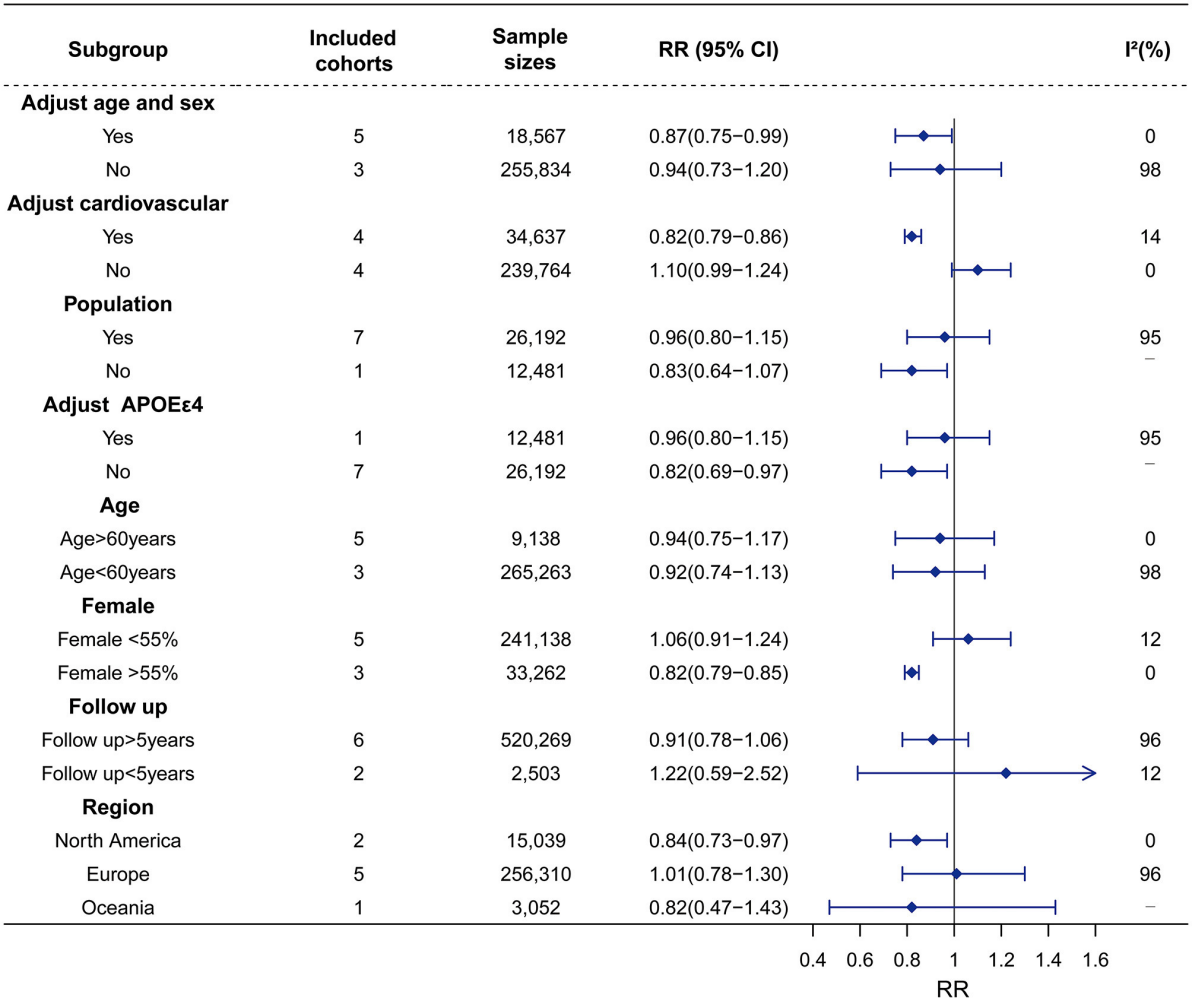
Association of Hypo with risk of dementia in the longitudinal study. Subgroup analyses indicated that the significance of the primary result was not altered by adjustment factor (no adjust age, sex, vascular factor, and adjust APOEε4), source of cohorts (population), age (>60 years vs. < 60 years), female (< 55%), follow up and region of Europe and Oceania. However, the pooled results tend to be reduced dementia risk in adjustment factors (adjust age, sex, vascular factor, and no adjust APOEε4), female (>55%), region of North America. The heterogeneity was reduced in the sub-analysis of adjustment factors (vascular factor), and female (>55% vs. < 55%). RR, relative risk; CI, confidence interval; AD, Alzheimer's disease; Hypo, hypothyroidism; *APOE*ε*4*, apolipoprotein E gene isoform 4.

Similarly, seven studies on Hypo with 27,441 participants were included. Hypo was not associated with the risk of all-cause dementia (RR = 0.93, 95% CI = 0.80–1.08, I^2^ = 94%). In the subgroup analysis according to subtypes of dementia, Hypo was associated with a decreased risk of AD (RR = 0.87, 95% CI = 0.80–0.96, I^2^ = 0) rather than VaD risk (RR = 1.18, 95% CI = 0.37–3.77, I^2^ = 84%) (Figure 2; Supplementary material 7). The high heterogeneity (I^2^ = 94%) was observed in the studies on the association between Hypo and dementia. Meta-regression revealed that adjusted vascular factors were the source of heterogeneity (*p* < 0.05) (Supplementary material 8). Sensitivity analysis showed robust effect estimates for dementia but not for AD (Supplementary material 9). Hypothyroidism is no association with AD risk after excluding one study (Thvilum et al., 2021) (RR = 1.02, 95% CI = 0.54–1.92) (Supplementary material 10). Subgroup analysis indicated that high heterogeneity was reduced by vascular factors and the percentage of females (Figure 4; Supplementary material 11).

No publication bias was revealed in the association between Hypo and dementia (*p* = 0.858, Supplementary material 12) or the association between Hypo and AD (*p* = 0.744, Supplementary material 12).

##### 3.2.1.3. Subclinical thyroid disease and dementia

We conducted two separate subgroup analyses of the included studies on Hype and Hypo according to whether the abnormal thyroid function status was clinical or subclinical. A meta-analysis of four studies with 28,997 individuals showed that patients with SHype had an increased risk of dementia (RR = 1.56, 95% CI = 1.26–1.93; I^2^ = 0%, Figure 2) compared to patients without SHype. The funnel plot for the studies on the association between SHype and ACD risk showed a symmetrical distribution, indicating no publication bias (*p* = 0.466).

Another meta-analysis of four studies with 20,594 individuals showed that patients with SHypo were not associated with the risk of ACD compared to patients without SHypo (RR = 0.84, 95% CI = 0.70–1.01; I^2^ = 15%, Figure 2). The results of the funnel plot (Supplementary material 13) and Egger test (Supplementary material 14) demonstrated that no publication bias in these four studies (*p* = 0.466). Additionally, the greater heterogeneity may be caused by different abnormal thyroid function statuses.

#### 3.2.2. Cross-sectional studies

As for cross-sectional studies, a meta-analysis of 2 studies with 1,163 individuals showed that the pooled RR of dementia reached 2.28 (95% CI = 0.63–8.27, I^2^ = 49%) in patients with Hype compared with those without Hype (Supplementary material 15). In addition, another meta-analysis of 2 studies with 975 individuals showed that Hypo was also not associated with the risk of ACD (RR = 0.83, 95% CI = 0.53–1.30, I^2^ = 0%), and then a meta-analysis of 2 studies with 2,110 individuals showed that Hypo was also not associated with the risk of cognitive impairment (RR = 1.43, 95% CI = 0.65–3.15, I^2^ = 83%) (Supplementary material 16).

## 4. Discussion

The results of our study showed that in longitudinal studies, Hype and SHype would increase the risk of ACD, whereas Hypo and SHypo were not associated with ACD risk; and both Hype and Hypo were not associated with ACD risk in cross-sectional studies.

### 4.1. Hype

Our analyses revealed that in longitudinal studies, Hype would increase the risk of ACD with moderate heterogeneity, but it was not associated with the risks of ACD subtypes (AD and VaD). The observed heterogeneity might be due to cardiovascular factors. We performed a meta-regression to explore whether cardiovascular factors were the source of heterogeneity, which yielded positive results. To eliminate the interference of cardiovascular factors, we conducted a subgroup analysis according to whether the study had adjustment for cardiovascular factors, which showed that Hype still significantly increased the risk of dementia after the adjustment. This further demonstrated that Hype was associated with dementia risk, independent of cardiovascular factors, which was validated by sensitivity analyses. This association was partly due to the decreased functional connectivity between the hippocampus and cortex (Zhang et al., 2014a). A second probable explanation was that Hype patients exhibited decreased gray matter volumes in cognition-related brain regions (Zhang et al., 2014b). A third explanation for this association was that Hype patients had a decreased concentration of glutamate in the posterior cingulate cortex, which might induce brain dysfunction (Liu et al., 2012). In addition, our subgroup analysis showed that SHype significantly increased the risk of dementia, which was in line with previous meta-analyses (Rieben et al., 2016; Tang et al., 2021). However, the existing studies do not provide an exact mechanism underlying the association between subclinical hyperthyroidism and dementia.

However, in cross-sectional studies, Hype was not associated with dementia risk, which was inconsistent with that from longitudinal studies. This result from cross-sectional studies should be interpreted with caution due to the limited sample size (only two studies included).

### 4.2. Hypo

Our meta-analysis showed that in longitudinal studies, Hypo was not associated with ACD, with high heterogeneity. Based on the results of meta-regression, the heterogeneity might come from adjusted confounders (cardiovascular factors and APOEε4), and the source of participants in cohorts. The heterogeneities were significantly reduced in the subgroup analyses according to whether the cardiovascular factors were adjusted and the female proportion (>55% vs. < 55%). Interestingly, the associations became significant when specific factors (cardiovascular factors and the female proportion) were included as covariates, suggesting the stratified or mediating effects might exist. Moreover, Hypo was negatively associated with AD. Sensitivity analysis showed the association between Hypo and AD became not significant after the exclusion of one study. In addition, we found that SHypo was not associated with the risk of dementia.

### 4.3. Strengths and limitations

The strengths of our meta-analysis should be mentioned. Firstly, only observational studies were included in our meta-analysis, interventional studies were excluded. Hence, all the included articles were studies of medium-to-high quality. Secondly, this meta-analysis did not show evident publication bias, indicating no small-study effects. Thirdly, since the longitudinal and cross-sectional studies were discussed separately in our meta-analyses, our results were more reliable with reduced bias. Fourthly, there were moderate-to-substantial heterogeneities in the main analyses. The possible sources of these heterogeneities were systematically evaluated using subgroup analyses. Lastly, our study is a comprehensive study. We explored the relationships of thyroid diseases with dementia as well as dementia subtypes. And we updated our data and included more studies.

This study also has some limitations. (1) The primary analysis showed significant heterogeneity. After carrying out subgroup analyses, the heterogeneity of results may be due to differences in sample size, methodological quality, demographic and ethno-racial characteristics, various covariate assessments, follow-up duration, subtypes of dementia, and methods for TSH measurement. Owing to inadequate information, it was difficult to analyze the heterogeneity further. (2) Studies focused on the relationships of thyroid cancer and thyroiditis with the risk of dementia are rare. Additional reliable evidence is needed for these relationships. (3) The associations identified by the analyses based on observational studies, but little is known about the causality. Randomized controlled trials are warranted in the future to test the roles of thyroid disease in preventing cognitive decline or dementia. (4) Due to the small scale of observational studies exploring the subtypes of dementia, it was difficult to conduct a meta-analysis of the relationships between thyroid diseases and the subtypes of dementia. More high-quality cohorts or nest case-control studies on the subtypes of dementia are warranted in the future.

## 5. Conclusion

Our systematic review and meta-analysis could provide evidence for positive associations of Hype and SHype with an increased risk of incident dementia. Hypo was found to reduce the risk of AD. Hence, Hype and SHype might be potentially modifiable causes of dementia, and enhanced screening services, timely intervention, and efficient treatment for Hype and SHype would reduce the incidence of dementia. In addition, more observational studies are urgently needed to explore the associations between thyroid diseases and the risk of dementia.

## Data availability statement

The original contributions presented in the study are included in the article/Supplementary material, further inquiries can be directed to the corresponding author.

## Author contributions

Concept, design, obtained funding, administrative, technical, and material support: LT. Drafting of the manuscript, statistical analysis, and chart design: L-YM and BZ. Acquisition, analysis, or interpretation of data, and critical revision of the manuscript for important intellectual content: all authors. All authors contributed to the article and approved the submitted version.

## References

[B1] Akintola A. A. Jansen S. W. van Bodegom D. van der Grond J. Westendorp R. G. de Craen A. J. . (2015). Subclinical hypothyroidism and cognitive function in people over 60 years: a systematic review and meta-analysis. Front. Aging Neurosci. 7, 150. 10.3389/fnagi.2015.00150 26321946 PMC4531303

[B2] Alzheimer's Association Report. (2021). 2021 Alzheimer's disease facts and figures. Alzheimer's Dementia J. Alzheimer's Assoc. 17, 327–406. 10.1002/alz.12328 33756057

[B3] Aubert C. E. Bauer D. C. da Costa B. R. Feller M. Rieben C. Simonsick E. M. . (2017). The association between subclinical thyroid dysfunction and dementia: The Health, Aging and Body Composition (Health ABC) Study. Clin Endocrinol 87, 617–626. 10.1111/cen.13458 28850708 PMC5658241

[B4] Bajaj S. Sachan S. Misra V. Varma A. Saxena P. (2014). Cognitive function in subclinical hypothyroidism in elderly. Indian J. Endocrinol. Metab. 18, 811–814. 10.4103/2230-8210.141355 25364675 PMC4192986

[B5] Benseñor I. M. Lotufo P. A. Menezes P. R. Scazufca M. (2010). Subclinical hyperthyroidism and dementia: the Sáo Paulo Ageing & Health Study (SPAH). BMC Public Health 10, 298. 10.1186/1471-2458-10-298 20515500 PMC2887825

[B6] Booth A. Clarke M. Dooley G. Ghersi D. Moher D. Petticrew M. . (2012). The nuts and bolts of PROSPERO: an international prospective register of systematic reviews. Syst. Rev. 1, 2. 10.1186/2046-4053-1-2 22587842 PMC3348673

[B7] Cárdenas-Ibarra L. Solano-Velázquez J. A. Salinas-Martínez R. Aspera-Ledezma T. D. Sifuentes-Martínez M.d.R. Villarreal-Pérez J. Z. (2008). Cross-sectional observations of thyroid function in geriatric Mexican outpatients with and without dementia. Arch. Gerontol. Geriatr. 46, 173–180. 10.1016/j.archger.2007.03.009 17512618

[B8] Ceresini G. Lauretani F. Maggio M. Ceda G. P. Morganti S. Usberti E. . (2009). Thyroid function abnormalities and cognitive impairment in elderly people: results of the Invecchiare in Chianti study. J. Am. Geriatr. Soc. 57, 89–93. 10.1111/j.1532-5415.2008.02080.x 19054181 PMC2631617

[B9] Chaimani A. Mavridis D. Salanti G. (2014). A hands-on practical tutorial on performing meta-analysis with Stata. Evid. Based Ment. Health 17, 111–116. 10.1136/eb-2014-101967 25288685 PMC13404023

[B10] Coory M. D. (2010). Comment on: heterogeneity in meta-analysis should be expected and appropriately quantified. Int. J. Epidemiol. 39, 932. 10.1093/ije/dyp157 19349478

[B11] De Jong F. J. Den Heijer T. Visser T. J. De Rijke Y. B. Drexhage H. A. Hofman A. . (2006). Thyroid hormones, dementia, and atrophy of the medial temporal lobe. J. Clin. Endocrinol. Metab. 91, 2569–2573. 10.1210/jc.2006-0449 16636121

[B12] De Jongh R. T. Lips P. Van Schoor N. M. Rijs K. J. Deeg D. J. H. Comijs H. C. . (2011). Endogenous subclinical thyroid disorders, physical and cognitive function, depression, and mortality in older individuals. Eur. J. Endocrinol. 165, 545–554. 10.1530/EJE-11-0430 21768248

[B13] DerSimonian R. Laird N. (2015). Meta-analysis in clinical trials revisited. Contemp. Clin. Trials 45, 139–145. 10.1016/j.cct.2015.09.002 26343745 PMC4639420

[B14] Duval S. Tweedie R. (2000). Trim and fill: A simple funnel-plot-based method of testing and adjusting for publication bias in meta-analysis. Biometrics 56, 455–463. 10.1111/j.0006-341X.2000.00455.x 10877304

[B15] Fernández-Lamo I. Montero-Pedrazuela A. Delgado-García J. M. Guadaño-Ferraz A. Gruart A. (2009). Effects of thyroid hormone replacement on associative learning and hippocampal synaptic plasticity in adult hypothyroid rats. Eur. J. Neurosci. 30, 679–692. 10.1111/j.1460-9568.2009.06862.x 19686470

[B16] Folkestad L. Brandt F. Lillevang-Johansen M. Brix T. H. Hegedüs L. (2020). Graves' disease and toxic nodular goiter, aggravated by duration of hyperthyroidism, are associated with Alzheimer's and vascular dementia: a registry-based long-term follow-up of two large cohorts. Thyroid 30, 672–680. 10.1089/thy.2019.0672 31984866

[B17] Forti P. Olivelli V. Rietti E. Maltoni B. Pirazzoli G. Gatti R. . (2012). Serum thyroid-stimulating hormone as a predictor of cognitive impairment in an elderly cohort. Gerontology 58, 41–49. 10.1159/000324522 21430364

[B18] George K. M. Lutsey P. L. Selvin E. Palta P. Windham B. G. Folsom A. R. (2019). Association between thyroid dysfunction and incident dementia in the atherosclerosis risk in communities neurocognitive study. J. Endocrinol. Metab. 9, 82–89. 10.14740/jem588 32411312 PMC7223793

[B19] Higgins J. P. Thompson S. G. (2002). Quantifying heterogeneity in a meta-analysis. Stat. Med. 21, 1539–1558. 10.1002/sim.1186 12111919

[B20] Kalmijn S. Mehta K. M. Pols H. A. Hofman A. Drexhage H. A. Breteler M. M. (2000). Subclinical hyperthyroidism and the risk of dementia. The Rotterdam study. Clin Endocrinol 53, 733–737. 10.1046/j.1365-2265.2000.01146.x 11155096

[B21] Knopman D. S. DeKosky S. T. Cummings J. L. Chui H. Corey-Bloom J. Relkin N. . (2001). Practice parameter: diagnosis of dementia (an evidence-based review). Report of the Quality Standards Subcommittee of the American Academy of Neurology. Neurology 56, 1143–1153. 10.1212/WNL.56.9.1143 11342678

[B22] Liu X. Bai Z. Liu F. Li M. Zhang Q. Song G. . (2012). Reduced posterior cingulate glutamate measured by magnetic resonance spectroscopy in hyperthyroidism. Neuro Endocrinol. Lett. 33, 626–630. 23160232

[B23] Manolis A. A. Manolis T. A. Melita H. Manolis A. S. (2020). Subclinical thyroid dysfunction and cardiovascular consequences: an alarming wake-up call? Trends Cardiovasc. Med. 30, 57–69. 10.1016/j.tcm.2019.02.011 30871865

[B24] Moher D. Liberati A. Tetzlaff J. Altman D. G. (2009). Preferred reporting items for systematic reviews and meta-analyses: the PRISMA statement. PLoS Med. 6, e1000097. 10.1371/journal.pmed.1000097 19621072 PMC2707599

[B25] O'Brien J. T. Thomas A. (2015). Vascular dementia. Lancet 386, 1698–1706. 10.1016/S0140-6736(15)00463-8 26595643

[B26] Park Y. J. Lee E. J. Lee Y. J. Choi S. H. Park J. H. Lee S. B. . (2010). Subclinical hypothyroidism (SCH) is not associated with metabolic derangement, cognitive impairment, depression or poor quality of life (QoL) in elderly subjects. Arch. Gerontol. Geriatr. 50, e68–73. 10.1016/j.archger.2009.05.015 19545916

[B27] Parsaik A. K. Singh B. Roberts R. O. Pankratz S. Edwards K. K. Geda Y. E. . (2014). Hypothyroidism and risk of mild cognitive impairment in elderly persons: a population-based study. JAMA Neurol. 71, 201–207. 10.1001/jamaneurol.2013.5402 24378475 PMC4136444

[B28] Pasqualetti G. Pagano G. Rengo G. Ferrara N. Monzani F. (2015). Subclinical hypothyroidism and cognitive impairment: systematic review and meta-analysis. J. Clin. Endocrinol. Metab. 100, 4240–4248. 10.1210/jc.2015-2046 26305618

[B29] Rieben C. Segna D. da Costa B. R. Collet T. H. Chaker L. Aubert C. E. . (2016). Subclinical thyroid dysfunction and the risk of cognitive decline: a meta-analysis of prospective cohort studies. J. Clin. Endocrinol. Metab. 101, 4945–4954. 10.1210/jc.2016-2129 27689250 PMC6287525

[B30] Seagroatt V. Stratton I. (1998). Bias in meta-analysis detected by a simple, graphical test. Test had 10% false positive rate. BMJ 316, 470–471. 9492688 PMC2665628

[B31] Sorbi S. Hort J. Erkinjuntti T. Fladby T. Gainotti G. Gurvit H. . (2012). EFNS-ENS guidelines on the diagnosis and management of disorders associated with dementia. Eur. J. Neurol. 19, 1159–1179. 10.1111/j.1468-1331.2012.03784.x 22891773

[B32] Stang A. (2010). Critical evaluation of the Newcastle-Ottawa scale for the assessment of the quality of nonrandomized studies in meta-analyses. Eur. J. Epidemiol. 25, 603–605. 10.1007/s10654-010-9491-z 20652370

[B33] Stroup D. F. Berlin J. A. Morton S. C. Olkin I. Williamson G. D. Rennie D. . (2000). Meta-analysis of observational studies in epidemiology: a proposal for reporting. Meta-analysis Of Observational Studies in Epidemiology (MOOSE) group. JAMA 283, 2008–2012. 10.1001/jama.283.15.2008 10789670

[B34] Tang X. Song Z. H. Wang D. Yang J. Augusto Cardoso M. Zhou J. B. . (2021). Spectrum of thyroid dysfunction and dementia: a dose-response meta-analysis of 344,248 individuals from cohort studies. Endocr. Connect 10, 410–421. 10.1530/EC-21-0047 33875615 PMC8111311

[B35] Taylor P. N. Albrecht D. Scholz A. Gutierrez-Buey G. Lazarus J. H. Dayan C. M. . (2018). Global epidemiology of hyperthyroidism and hypothyroidism. Nat. Rev. Endocrinol. 14, 301–316. 10.1038/nrendo.2018.18 29569622

[B36] Thvilum M. Brandt F. Lillevang-Johansen M. Folkestad L. Brix T. H. Hegedüs L. (2021). Increased risk of dementia in hypothyroidism: a Danish nationwide register-based study. Clin. Endocrinol. 94, 1017–1024. 10.1111/cen.14424 33484007

[B37] Vadiveloo T. Donnan P. T. Cochrane L. Leese G. P. (2011). The thyroid epidemiology, audit, and research study (TEARS): morbidity in patients with endogenous subclinical hyperthyroidism. J. Clin. Endocrinol. Metab. 96, 1344–1351. 10.1210/jc.2010-2693 21346066

[B38] Xu W. Tan C. C. Zou J. J. Cao X. P. Tan L. (2020). Sleep problems and risk of all-cause cognitive decline or dementia: an updated systematic review and meta-analysis. J. Neurol. Neurosurg. Psychiatry 91, 236–244. 10.1136/jnnp-2019-321896 31879285 PMC7035682

[B39] Yeap B. B. Alfonso H. Chubb S. A. Puri G. Hankey G. J. Flicker L. . (2012). Higher free thyroxine levels predict increased incidence of dementia in older men: the Health in Men Study. J. Clin. Endocrinol. Metab. 97, E2230–2237. 10.1210/jc.2012-2108 22977271

[B40] Zeng X. Zhang Y. Kwong J. S. Zhang C. Li S. Sun F. . (2015). The methodological quality assessment tools for preclinical and clinical studies, systematic review and meta-analysis, and clinical practice guideline: a systematic review. J. Evid. Based Med. 8, 2–10. 10.1111/jebm.12141 25594108

[B41] Zhang W. Liu X. Zhang Y. Song L. Hou J. Chen B. . (2014a). Disrupted functional connectivity of the hippocampus in patients with hyperthyroidism: evidence from resting-state fMRI. Eur. J. Radiol. 83, 1907–1913. 10.1016/j.ejrad.2014.07.003 25082476

[B42] Zhang W. Song L. Yin X. Zhang J. Liu C. Wang J. . (2014b). Grey matter abnormalities in untreated hyperthyroidism: a voxel-based morphometry study using the DARTEL approach. Eur. J. Radiol. 83, e43–48. 10.1016/j.ejrad.2013.09.019 24161779

