# Supplementary material for: Association of thyroid disease with risks of dementia and cognitive impairment: A meta-analysis and systematic review
Source: Front Aging Neurosci. 2023 Mar 13;15:1137584. doi: 10.3389/fnagi.2023.1137584 (PMC10040782; doi:10.3389/fnagi.2023.1137584)

***Supplementary Material***

**The Association Between Thyroid Disea­­se with Risk of Dementia and Cognitive Impairment: A Meta-Analysis and Systematic Review**

**Content:**

[Supplemental file-2: Meta-analysis for hyperthyroidism with the combined risk of dementia, AD, and VaD in the cohort study 5](#_Toc128348417)

[Supplemental file-3: Meta-regression analyses of the association between hyperthyroidism and dementia 7](#_Toc128348418)

[Supplemental file-4: Results of the sensitivity analyses of hyperthyroidism for dementia: 8](#_Toc128348419)

[Supplemental file-5: Subgroup analyses for the association between hyperthyroidism and dementia 10](#_Toc128348420)

[Supplemental file-6: Funnel plots 14](#_Toc128348421)

[Supplemental file-7: Meta-analysis for hypothyroidism with the combined risk of dementia, AD, VaD, and cognitive impairment in the cohort studies 16](#_Toc128348422)

[Supplemental file-8: Meta-regression analyses of the association between hypothyroidism and Dementia 18](#_Toc128348423)

[Supplemental file-9: Results of the sensitivity analyses of Hypothyroidism 19](#_Toc128348424)

[sensitivity analyses for dementia: 19](#_Toc128348425)

[Supplemental file-10: Meta-analysis for hypothyroidism with the combined risk of Alzheimer's disease in excluding one study 21](#_Toc128348426)

[Supplemental file-11: Subgroup analyses for the association between hypothyroidism and dementia in the cohort studies 22](#_Toc128348427)

[Supplemental file-12: Funnel plots of hypothyroidism 26](#_Toc128348428)

[Supplement file-13: Meta-analysis for subclinical thyroid disease with the combined risk of dementia in cohort studies 28](#_Toc128348429)

[Supplemental file-14: Funnel plots of subclinical thyroid disease 29](#_Toc128348430)

[Supplemental file-15: Meta-analysis for hyperthyroidism with the combined risk of dementia in the cross-sectional studies 31](#_Toc128348431)

[Supplemental file-16: Meta-analysis for hypothyroidism with the combined risk of cognitive impairment and dementia in Cross-sectional studies 31](#_Toc128348432)

**Supplemental file-1: Newcastle-Ottawa Scale of Cohort study and Case-control studies included in the meta-analysis and systematic review**

**A**

| **First Author** | **Year** | **Cohort Name** | **Selection** | **Comparability** | **Outcome** |
| --- | --- | --- | --- | --- | --- |
| **Aubert** | 2017 | Health ABC Study | ★★★★ | ★★ | ★★★ |
| **George** | 2019 | ARIC | ★★★★ | ★★ | ★★★ |
| **Kalmijn** | 2000 | TRS | ★★★★ | ★★ | ★★ |
| **De Jong** | 2006 | TRSS | ★★★★ | ★ | ★★★ |
| **Vadiveloo** | 2011 | TEARS | ★★★★ | ★★ | ★★★ |
| **Forti** | 2012 | CSBA | ★★★★ | ★ | ★★ |
| **Thvilum** | 2021 | DNPR | ★★★★ | ★ | ★★★ |
| **Thvilum** | 2021 | OPENTHYRO | ★★★★ | ★ | ★★★ |
| **De Jongh** | 2011 | LASA | ★★★★ | ★★ | ★★★ |
| **Yeap** | 2012 | HIMS | ★★★★ | ★★ | ★★★ |
| **Folkestad** | 2020 | DNPR | ★★★★ | ★★ | ★★ |
| **Folkestad** | 2020 | OPENTHYRO | ★★★★ | ★★ | ★★ |

Health ABC Study: The Health, Aging and Body Composition Study; ARIC: Atherosclerosis Risk in Communities Neurocognitive Study; TRS: the Rotterdam Study; TRSS: the Rotterdam Scan Study; TEARS: The Thyroid Epidemiology, Audit, and Research Study; CSBA: the Conselice Study of Brain Ageing; DNPR: Danish National Patient Registry; OPENTHYRO: Odense Patient data Explorative Network Thyroid Status and Register Outcomes; LASA: the Longitudinal Aging Study Amsterdam; HIMS: Health in Men Study;

**B**

**Agency for Healthcare Research and Quality of Cross-sectional studies included in the meta-analysis and systematic review**

| **Study** | **items** | | | | | | | | | | | **Quality score** |
| --- | --- | --- | --- | --- | --- | --- | --- | --- | --- | --- | --- | --- |
|  | **①** | **②** | **③** | **④** | **⑤** | **⑥** | **⑦** | **⑧** | **⑨** | **⑩** | **⑪** |  |
| **Bajaj (2014)** | Y | Y | Y | Y | N | Y | Y | Y | UC | Y | UC | 9 |
| **Ca´rdenasIba (2006)** | Y | Y | Y | Y | N | Y | UC | Y | Y | Y | Y | 10 |
| **Park （2009）** | Y | Y | Y | Y | N | Y | Y | N | Y | UC | N | 8 |
| **Benseñor （2010）** | Y | Y | Y | Y | N | Y | Y | Y | Y | Y | N | 10 |
| **Parsaik （2014）** | Y | Y | Y | Y | N | Y | Y | Y | Y | Y | Y | 11 |
| 1. Define the source of information (survey, record review)②List inclusion and exclusion criteria for exposed and unexposed subjects (cases and controls) or refer to previous publications ③Indicate time period used for identifying patients④Indicate whether or not subjects were consecutive if not population-based ⑤Indicate if evaluators of subjective components of the study were masked to other aspects of the status of the participants ⑥Describe any assessments undertaken for quality assurance purposes (e.g., test/retest of primary outcome measurements)⑦Explain any patient exclusions from analysis ⑧Describe how confounding was assessed and/or controlled ⑨If applicable, explain how missing data were handled in the analysis⑩ Summarize patient response rates and completeness of data collection⑪Clarify what follow-up if any, was expected and the percentage of patients for which incomplete data or follow-up was obtained. Y: Yes; N: No; UC: unclear | | | | | | | | | | | | |

# Supplemental file-2: Meta-analysis for hyperthyroidism with the combined risk of dementia, AD, and VaD in the cohort study

**Dementia**

**
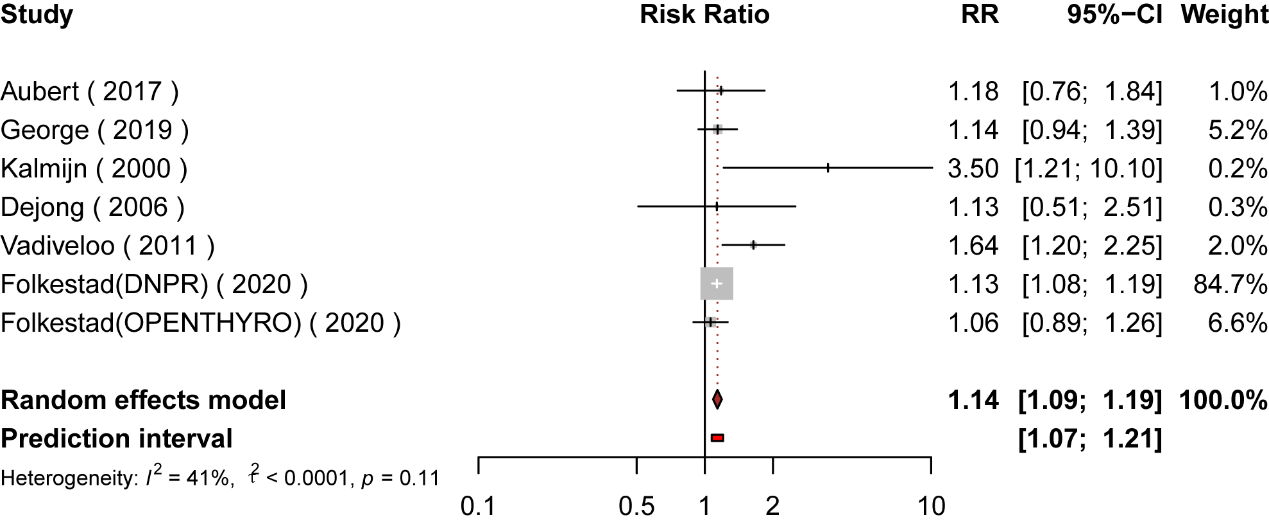
**

**AD**


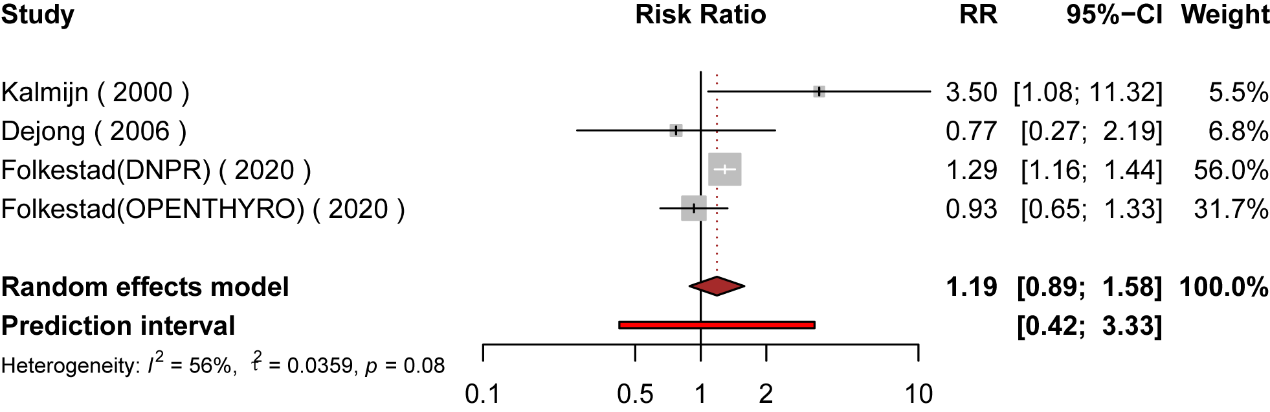


**VaD**


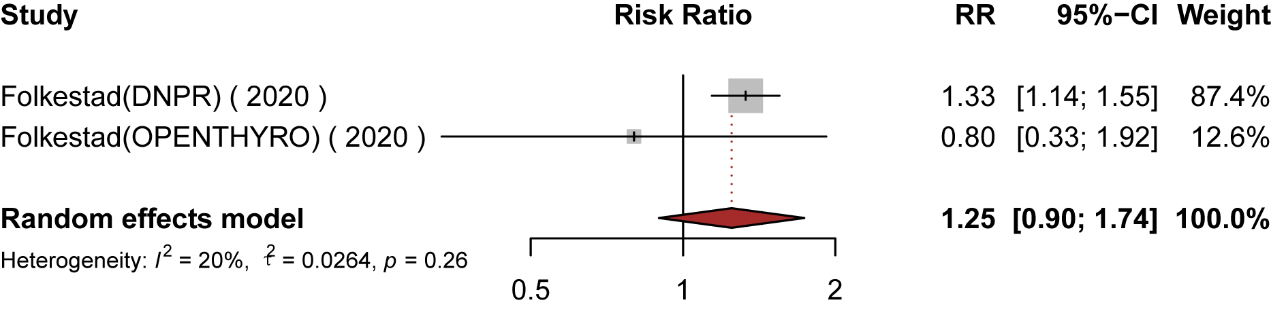


The size of the gray box presented the weighting of the study; the x-coordinate of the gray box indicated the risk estimate, and the black solid line indicated the corresponding 95% confidence interval (CI); the red dotted line indicated the combined RR value of the meta-analysis, and the red lozenge indicated the corresponding 95% CI. The legends applied to the following forest plots, as well.

AD: Alzheimer's disease, VaD: Vascular dementia

# Supplemental file-3: Meta-regression analyses of the association between hyperthyroidism and dementia

| **Variables*** | P value | Tau^2^ | I^2^ (%) | R^2^ (%) |
| --- | --- | --- | --- | --- |
| Adjust cardiovascular | 0.047 | 0 | 0 | 0 |
| Adjust APOEε4 | 0.770 | 0.0191 | 51.24 | 0 |
| Adjust age and sex | 0.272 | 0.0012 | 36.57 | 0 |
| Age（years） | 0.409 | 0 | 43.31 | 0 |
| Female (%) | 0.839 | 0.0162 | 51.09 | 0 |
| Follow up (years) | 0.114 | 0 | 15.62 | 0 |
| Source | 0.068 | 0 | 0 | 0 |
| Region | 0.770 | 0.0182 | 51.20 | 0 |

* Meta-regression was conducted on the study characteristics which were continuous variables; Tau^2^ indicated the estimate of between-study variance; I^2^ indicated residual variation due to heterogeneity; R^2^ indicated the proportion of between-study variance explained.

# Supplemental file-4: Results of the sensitivity analyses of hyperthyroidism for dementia:


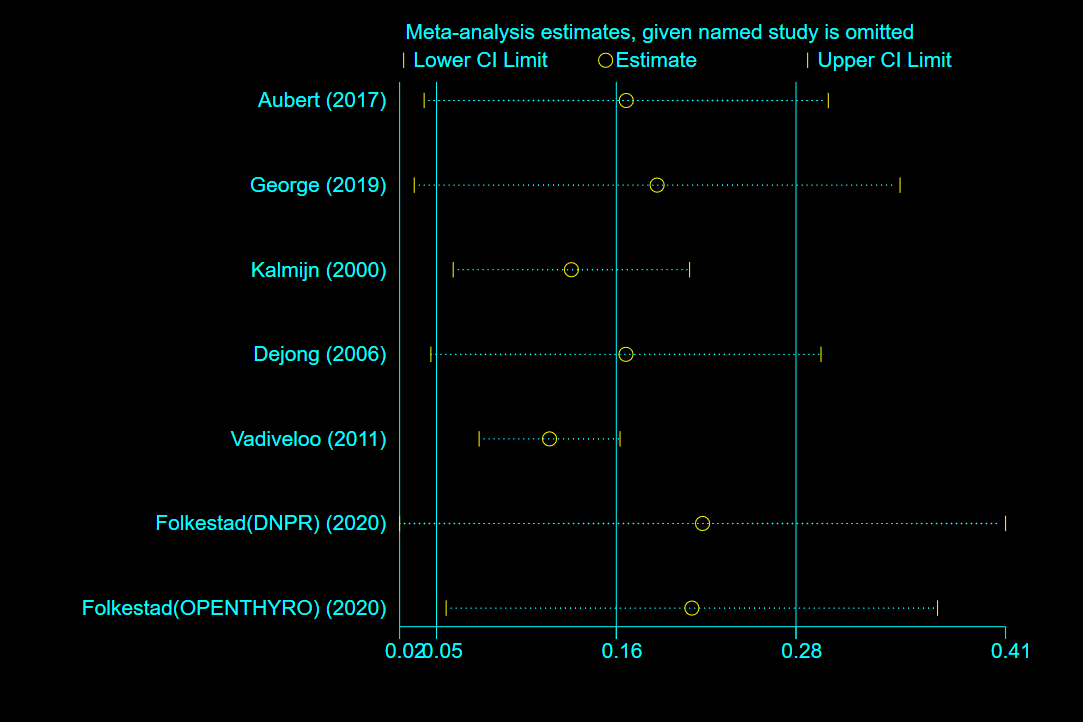


**Sensitivity analysis for AD:**


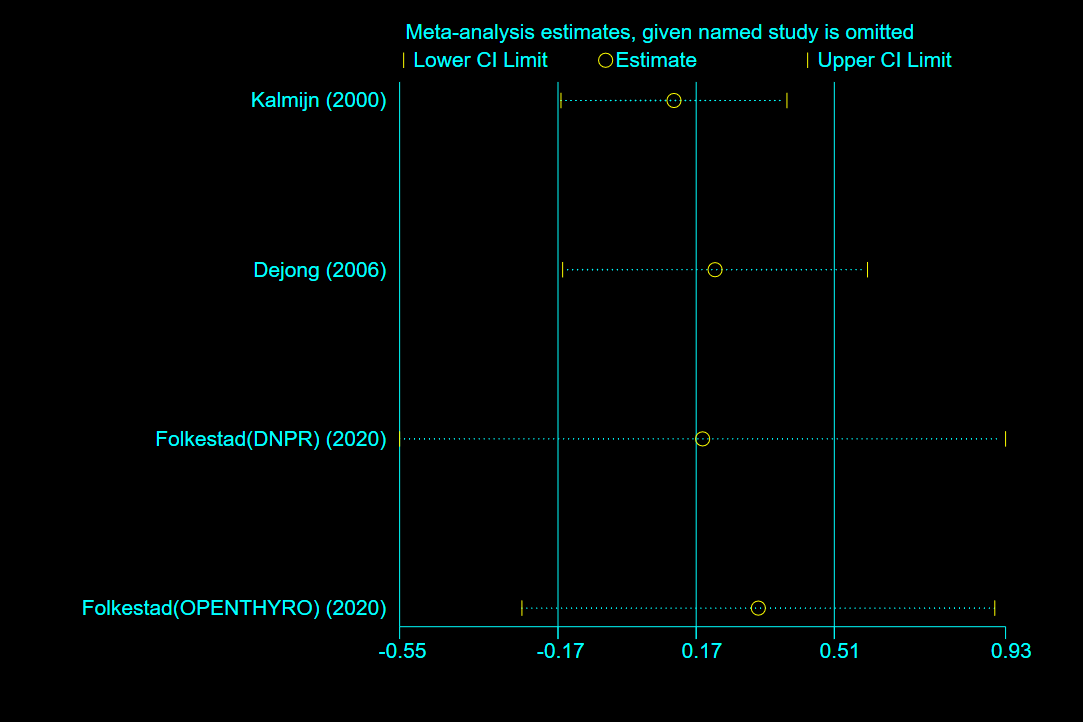


# Supplemental file-5: Subgroup analyses for the association between hyperthyroidism and dementia

**Adjusting for cardiovascular factors**


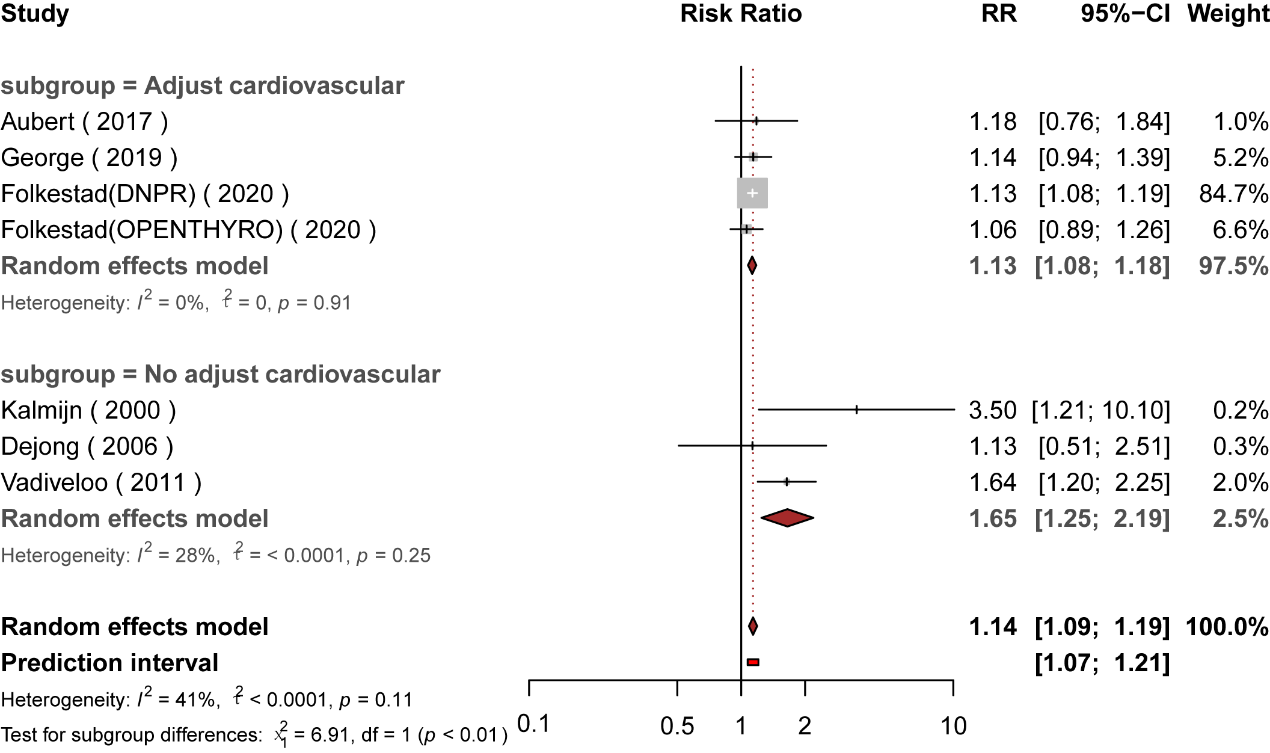


**Adjust for age and gender**


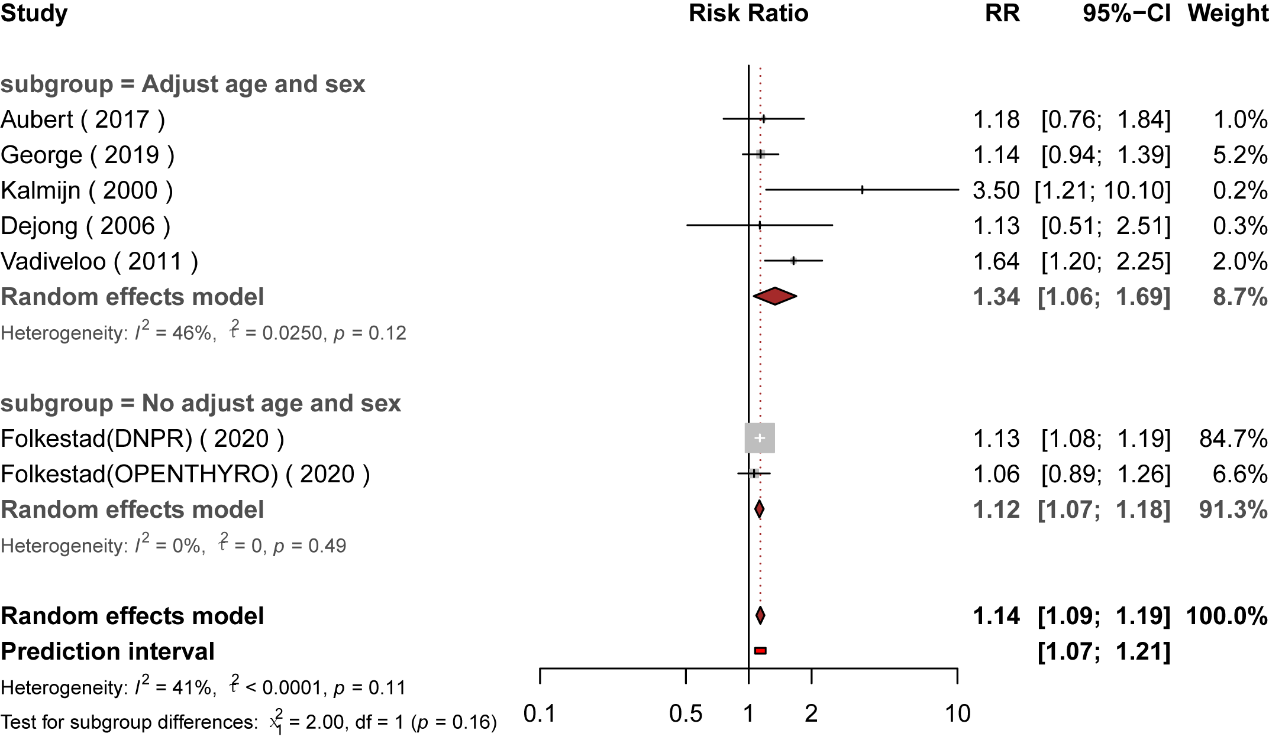


**Adjust for APOEε4**


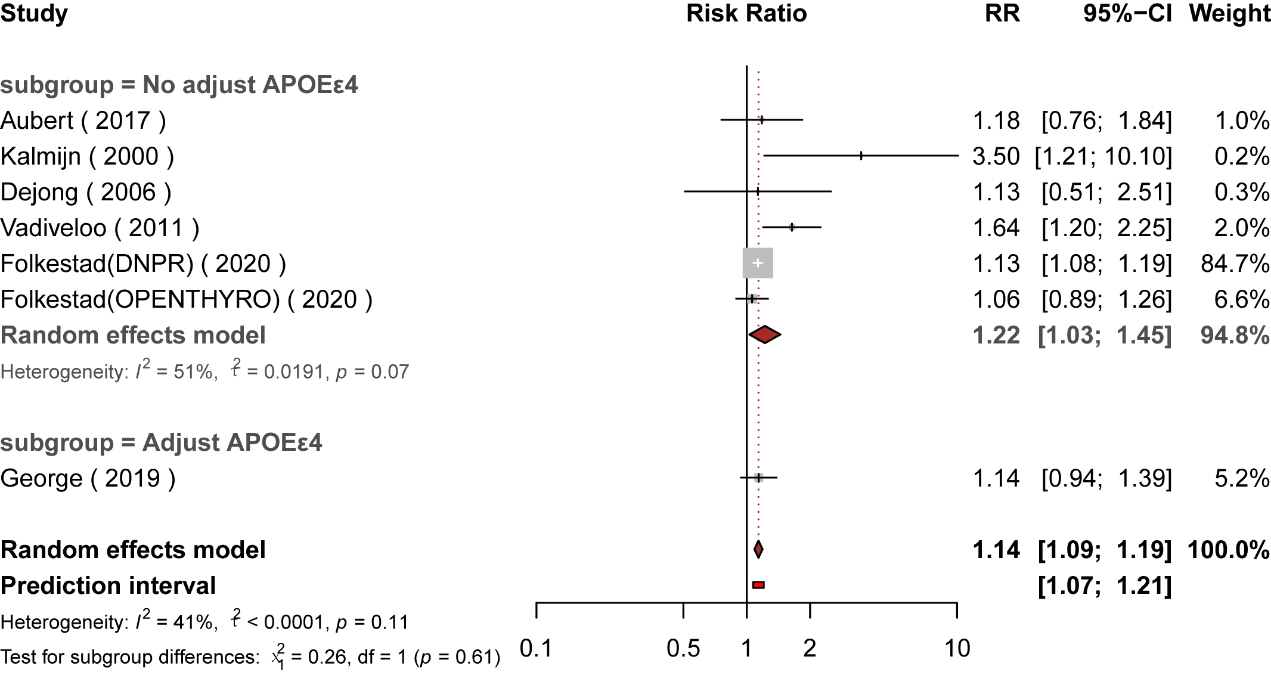


**Female**


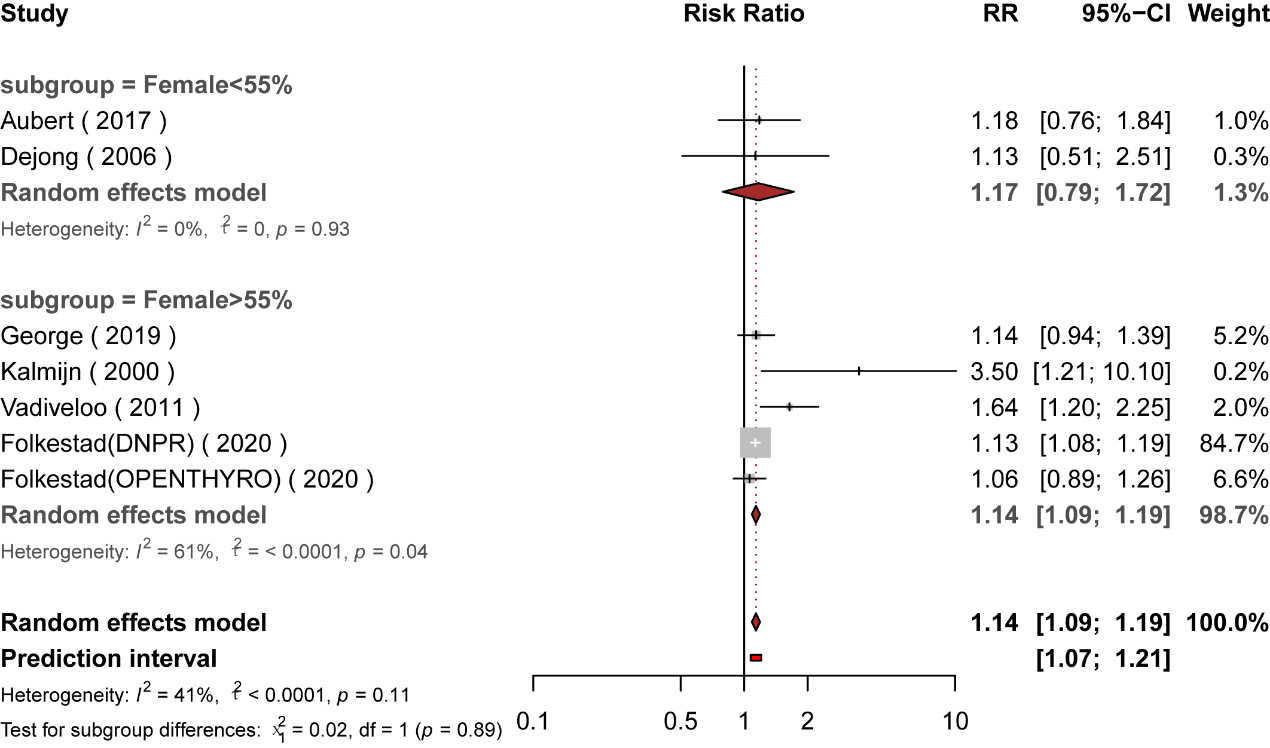


**Follow up**


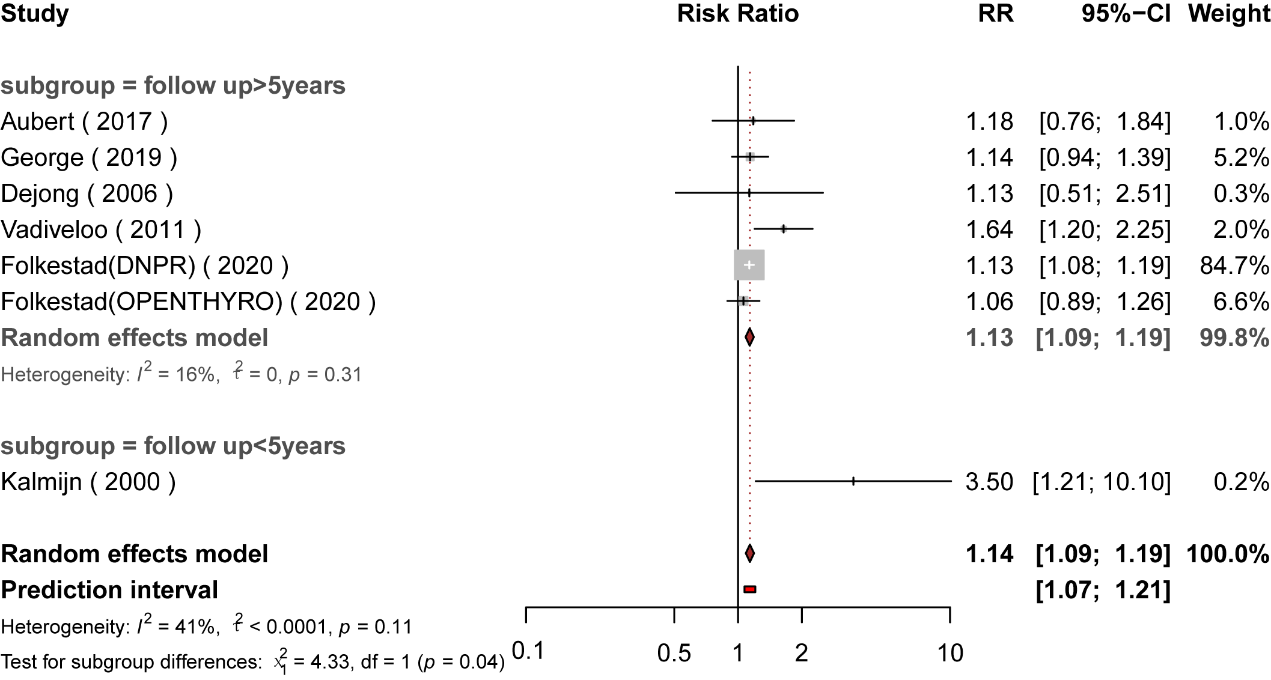


**Age**

**
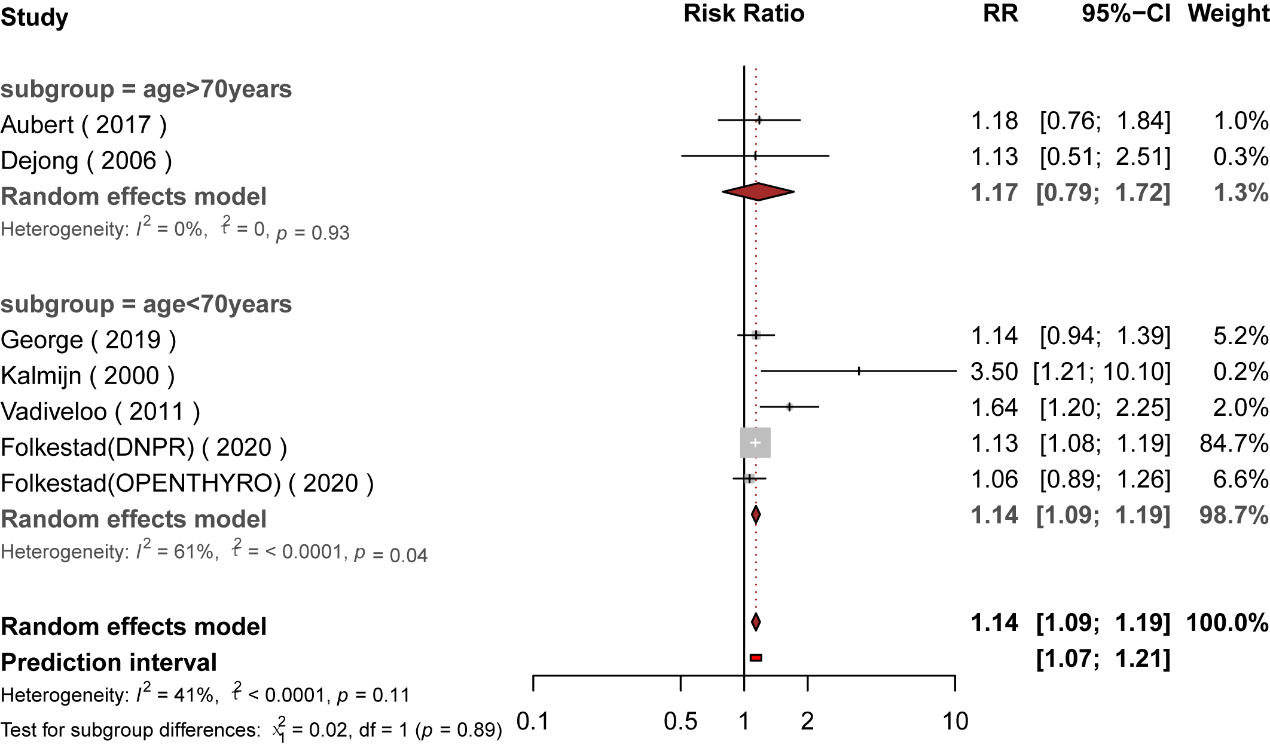
**

**Source**


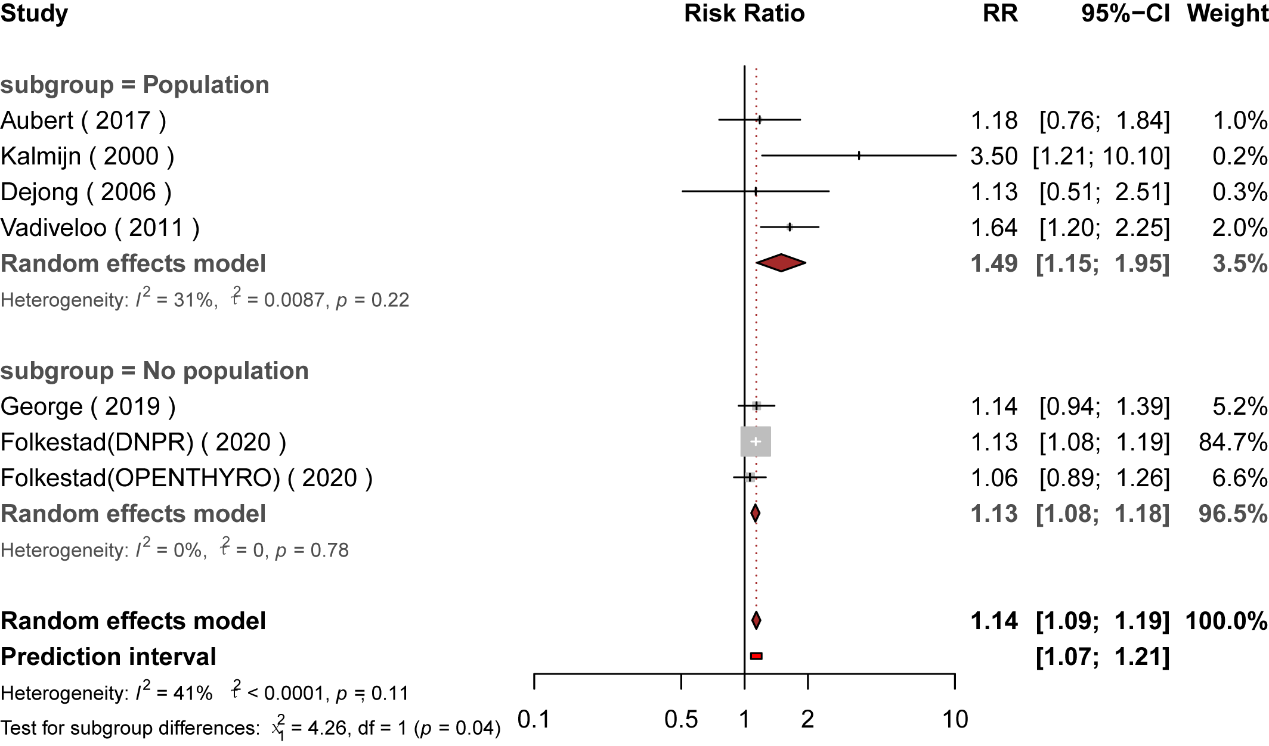


**Region**


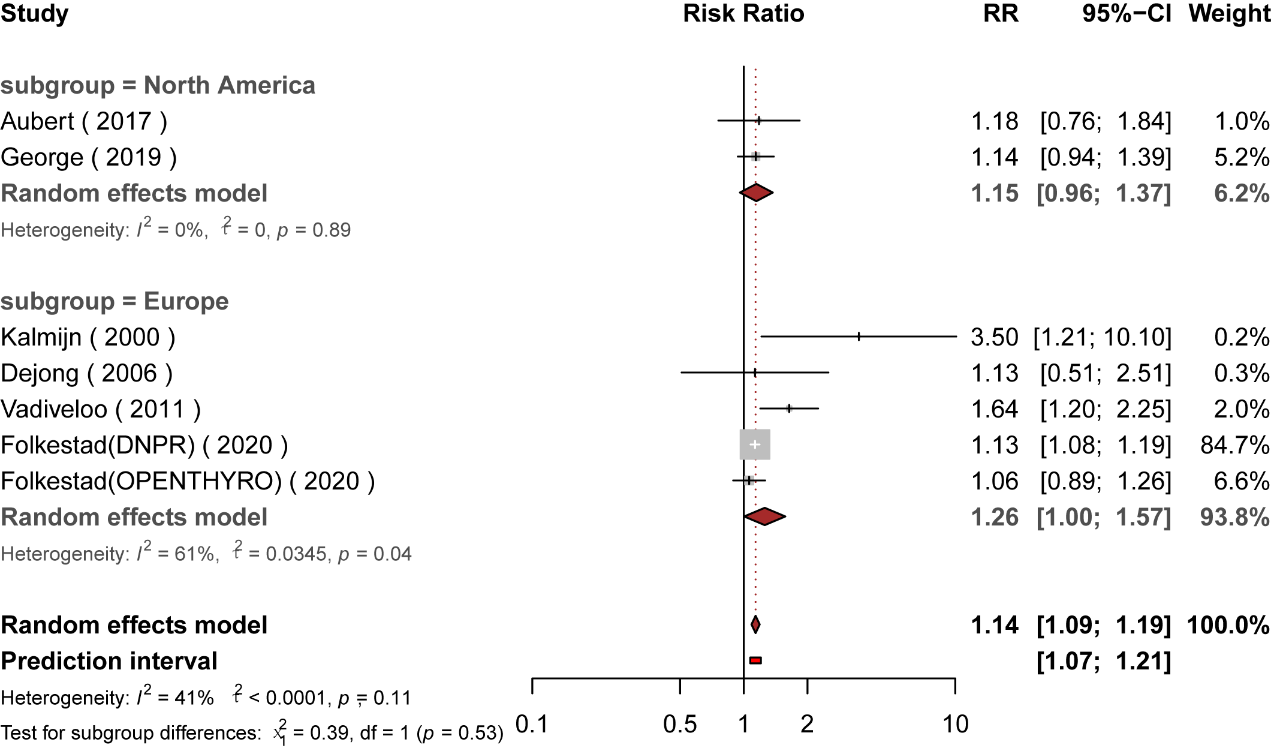


# Supplemental file-6: Funnel plots

**Hyperthyroidism and dementia**


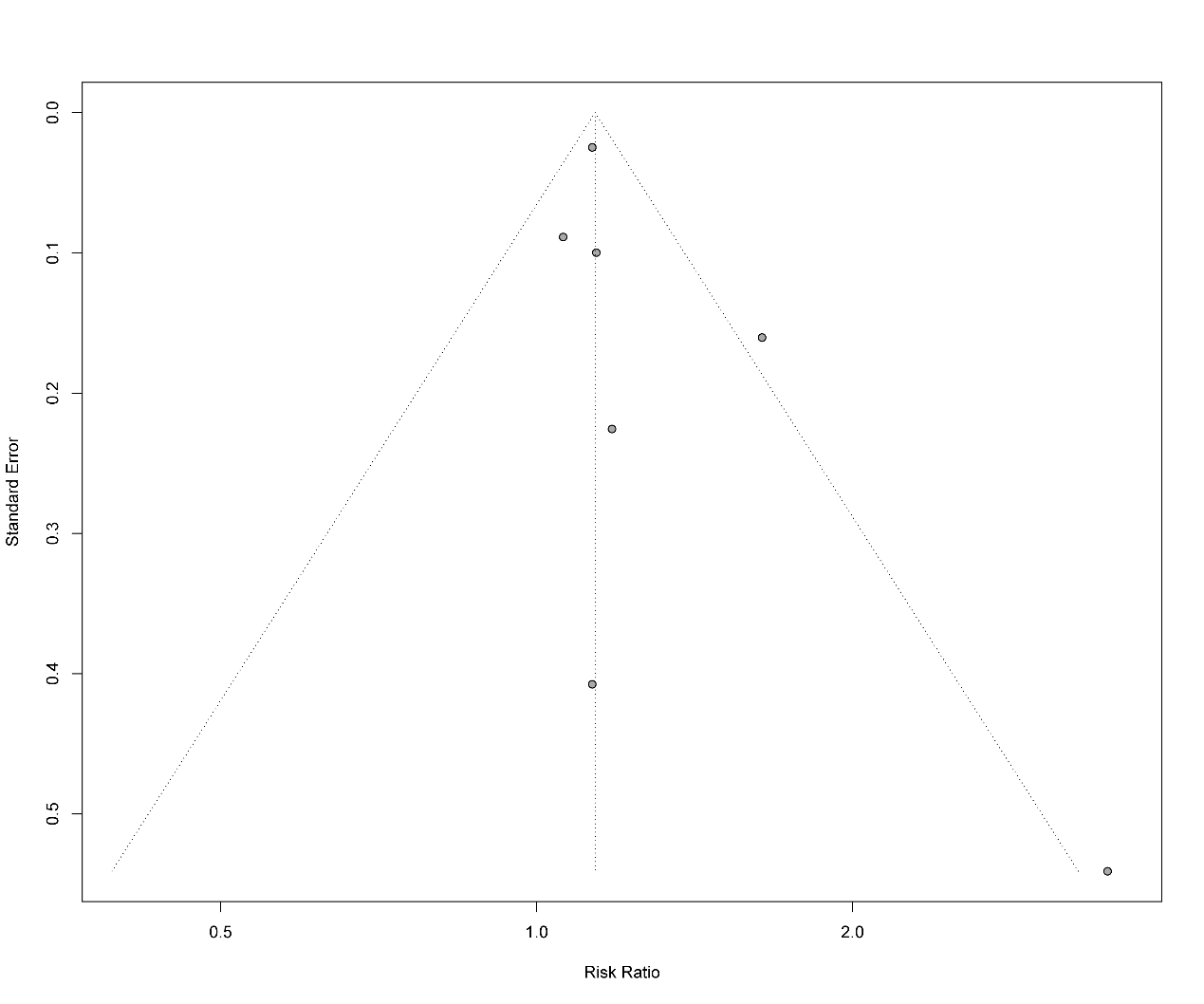


**Hyperthyroidism and Alzheimer's disease**


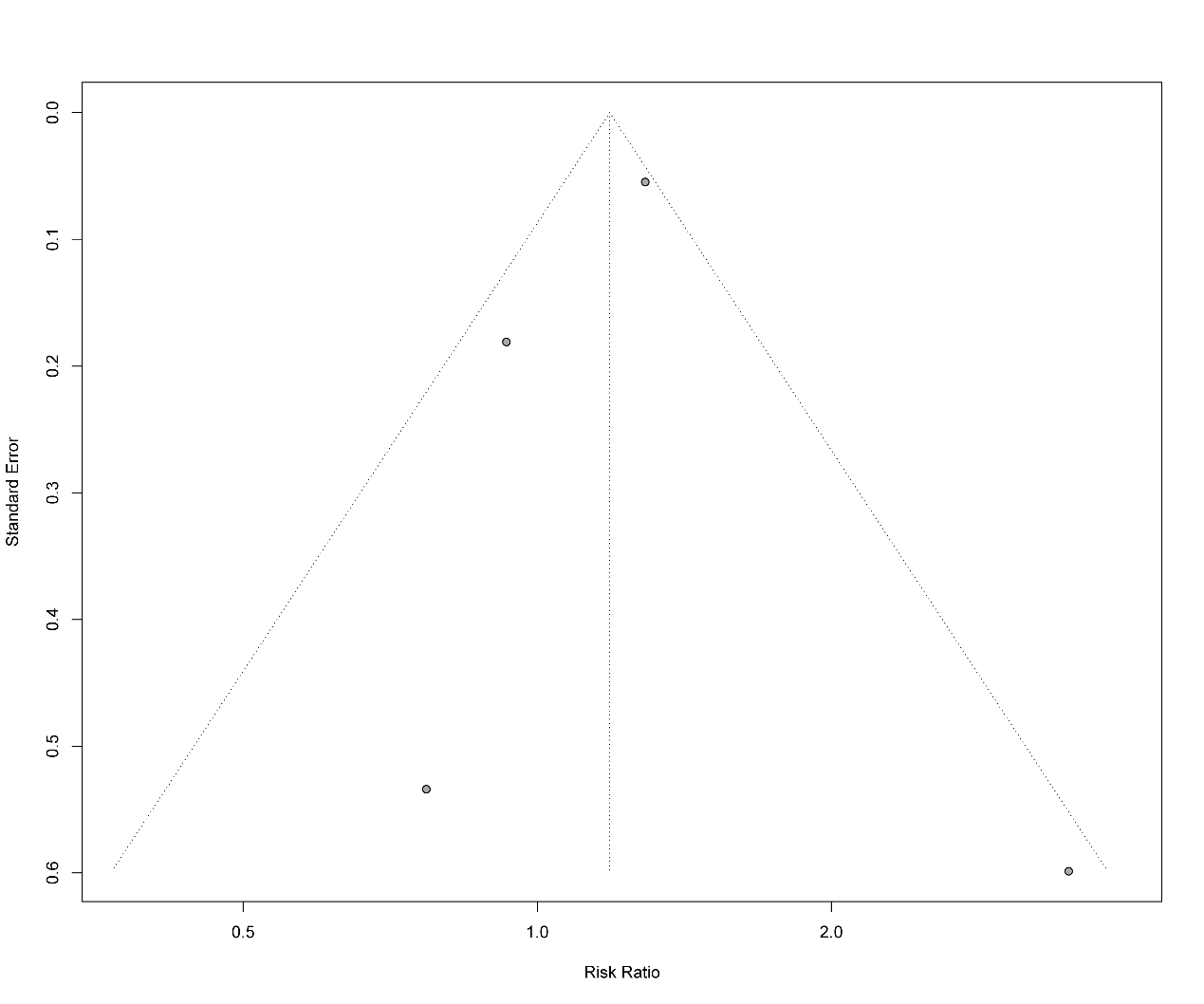


# Supplemental file-7: Meta-analysis for hypothyroidism with the combined risk of dementia, AD, VaD, and cognitive impairment in the cohort studies

**Dementia**


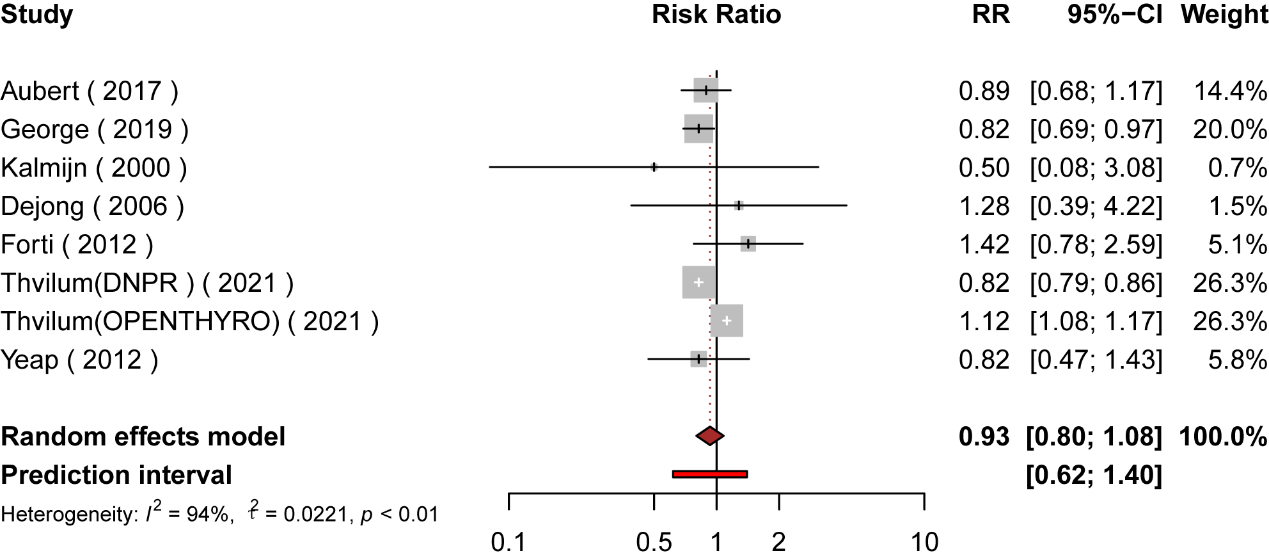


**AD**


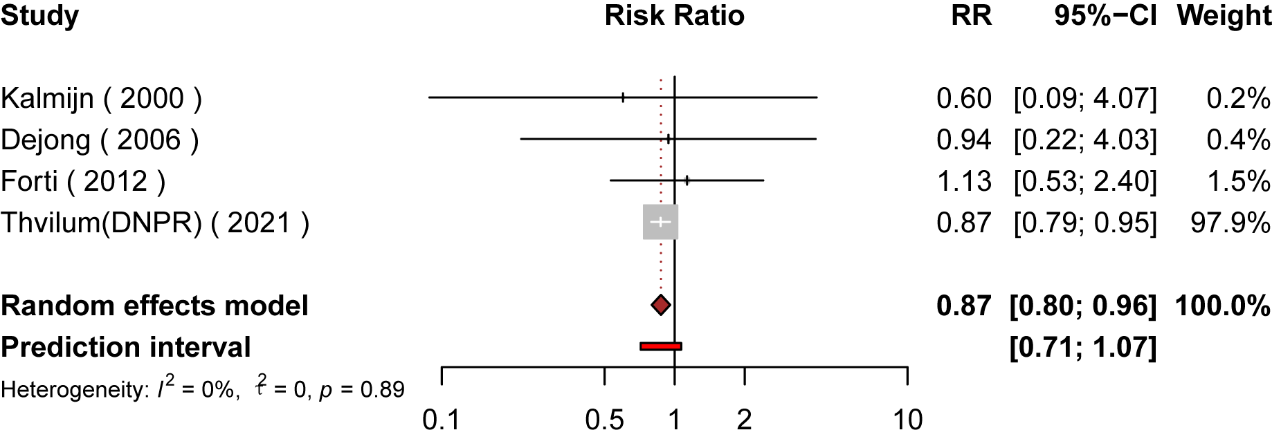


**VaD**


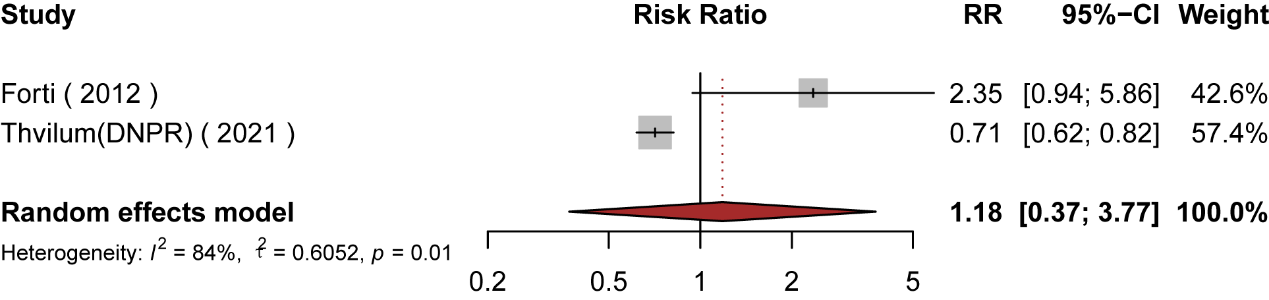


**Cognitive impairment**


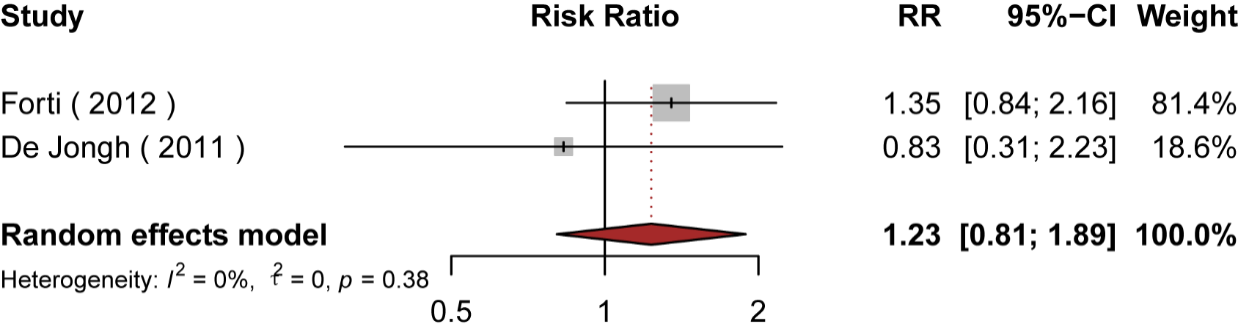


# Supplemental file-8: Meta-regression analyses of the association between hypothyroidism and Dementia

| **Variables*** | **P value** | **Tau^2^** | **I^2^ (%)** | **R^2^ (%)** |
| --- | --- | --- | --- | --- |
| **Adjust cardiovascular** | **0.000** | **0.0000** | **0.00** | **100.00** |
| **Adjust APOEε4** | **0.466** | **0.0250** | **94.63** | **-12.99** |
| **Adjust age and sex** | **0.878** | **0.0279** | **94.69** | **-25.95** |
| **Age（years）** | **0.859** | **0.0273** | **94.78** | **-23.35** |
| **Female (%)** | **0.717** | **0.0242** | **76.71** | **-9.22** |
| **Follow up(years)** | **0.368** | **0.0218** | **94.75** | **1.67** |
| **Population** | **0.466** | **0.0250** | **94.63** | **-12.99** |
| **Region** | **0.619** | **0.0243** | **94.66** | **-9.97** |

*** Meta-regression was conducted on the study characteristics which were continuous variables; Tau^2^ indicated the estimate of between-study variance; I^2^ indicated residual variation due to heterogeneity; R^2^ indicated the proportion of between-study variance explained.**

# Supplemental file-9: Results of the sensitivity analyses of Hypothyroidism

# sensitivity analyses for dementia:

**
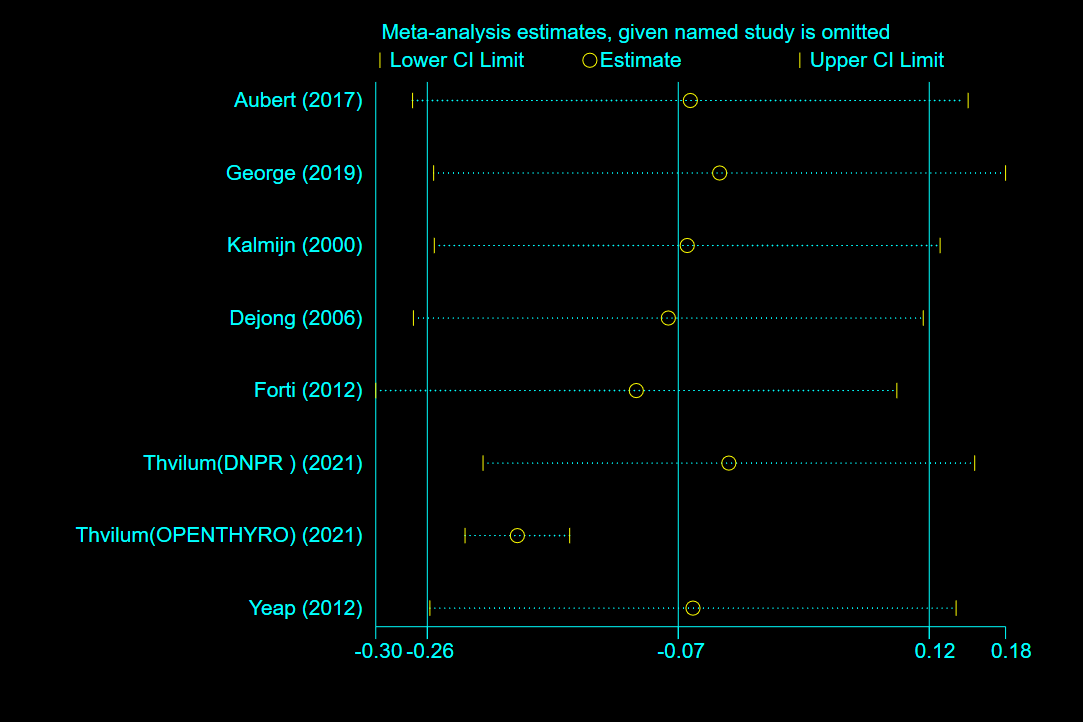
**

**sensitivity analyses for Alzheimer's disease:**

**
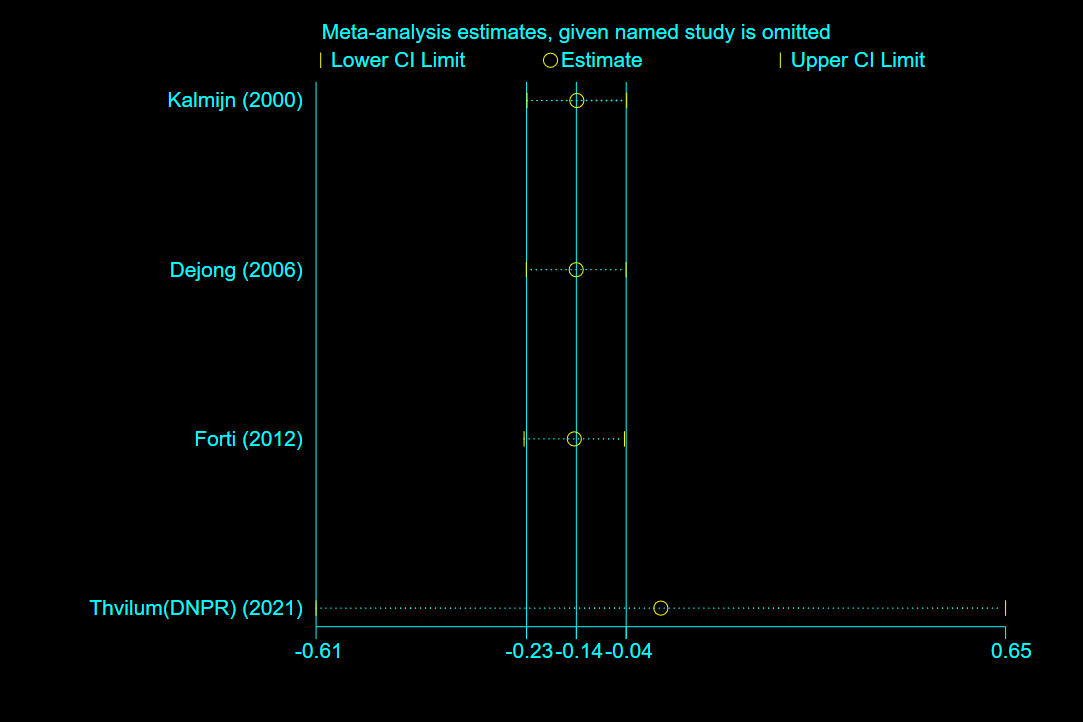
**

# Supplemental file-10: Meta-analysis for hypothyroidism with the combined risk of Alzheimer's disease in excluding one study

**AD**


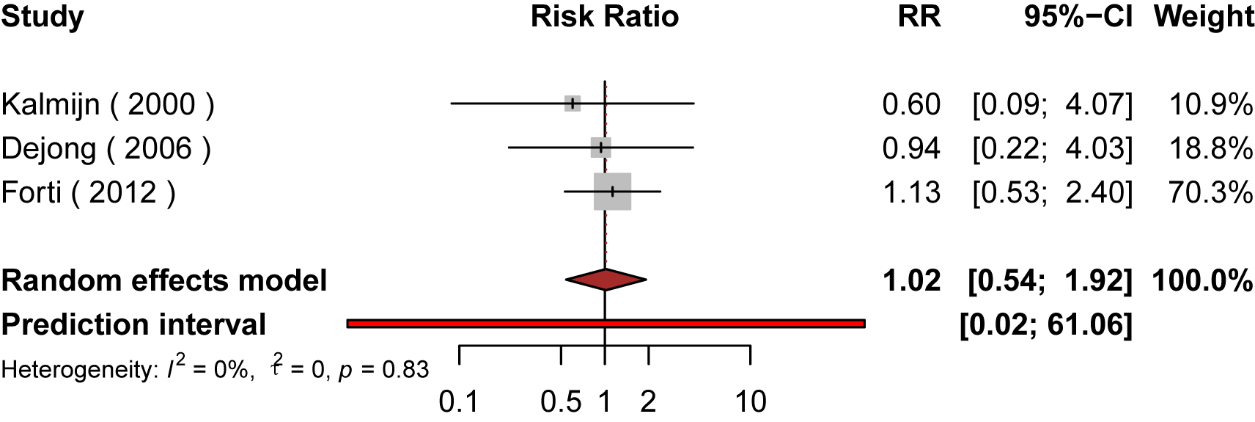


# Supplemental file-11: Subgroup analyses for the association between hypothyroidism and dementia in the cohort studies

**Adjust for cardiovascular risk factors**


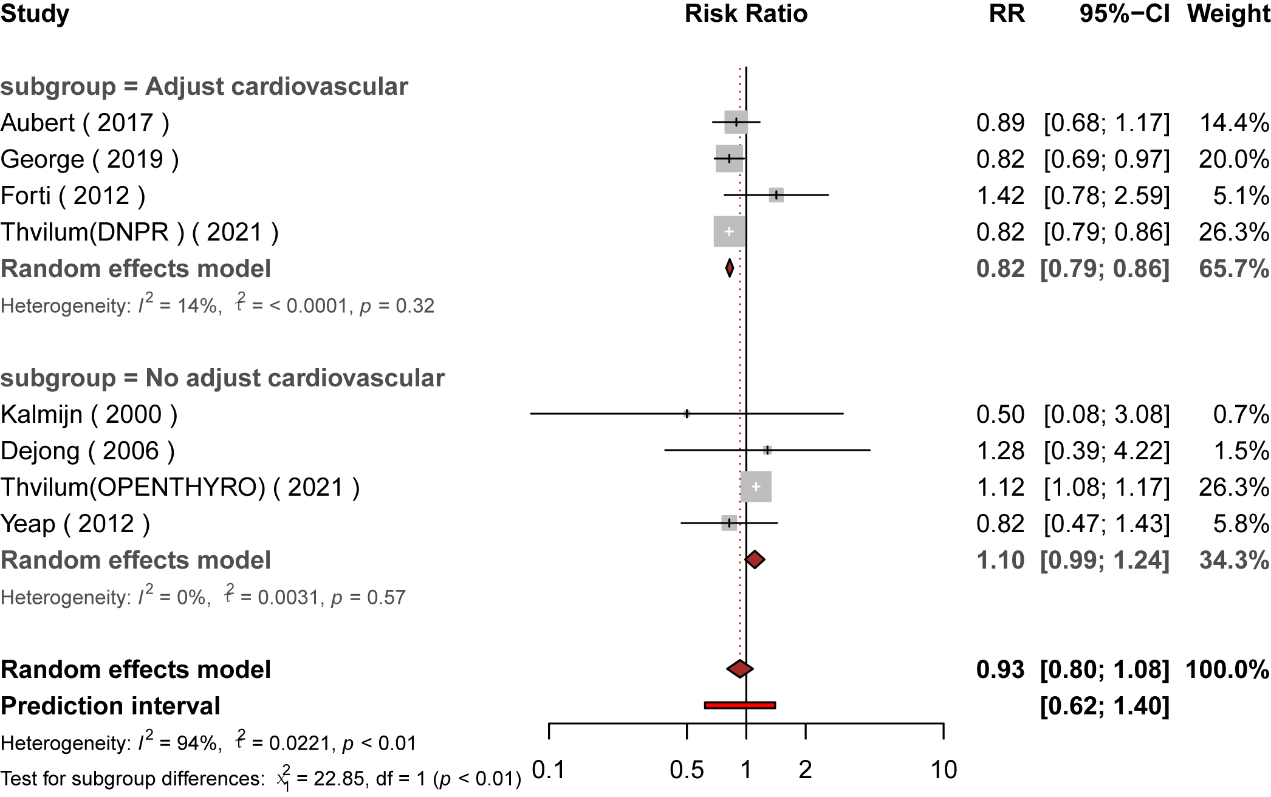


**Female**


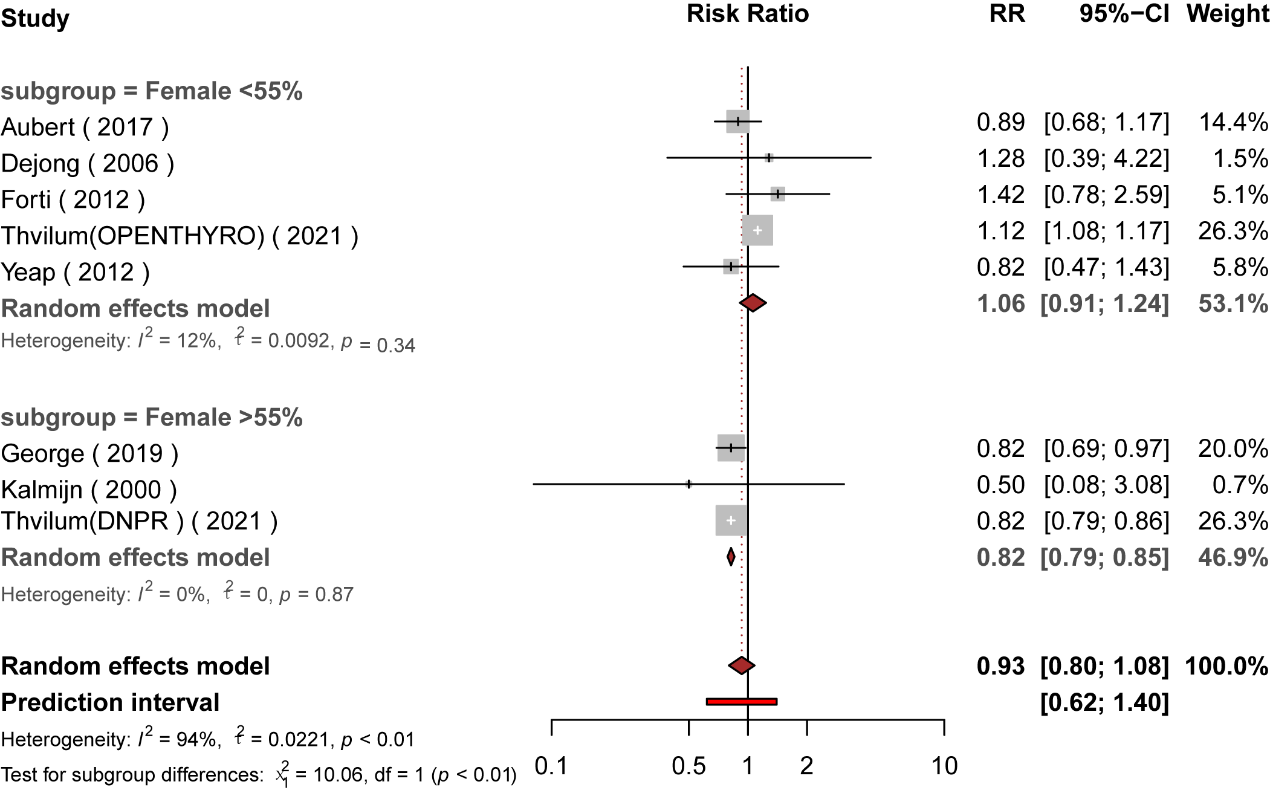


**Adjust for age and sex**


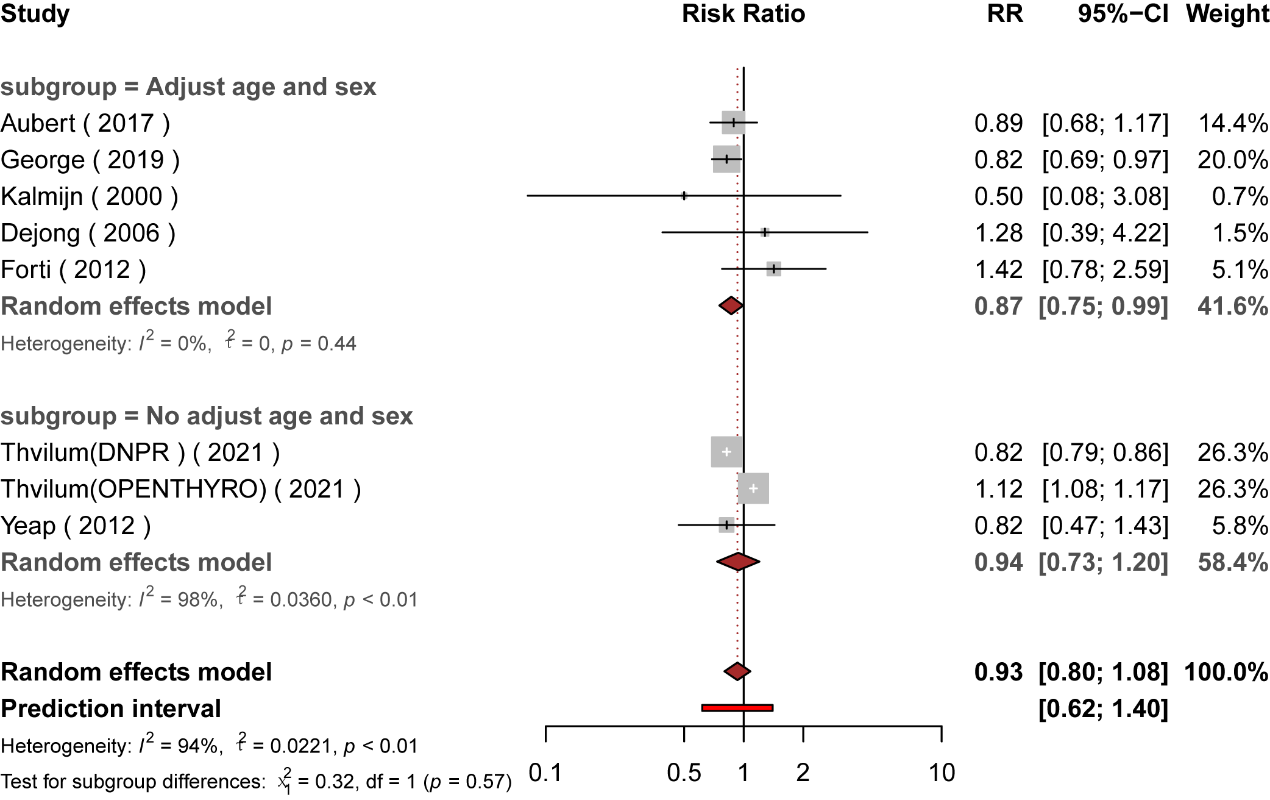


**Age**


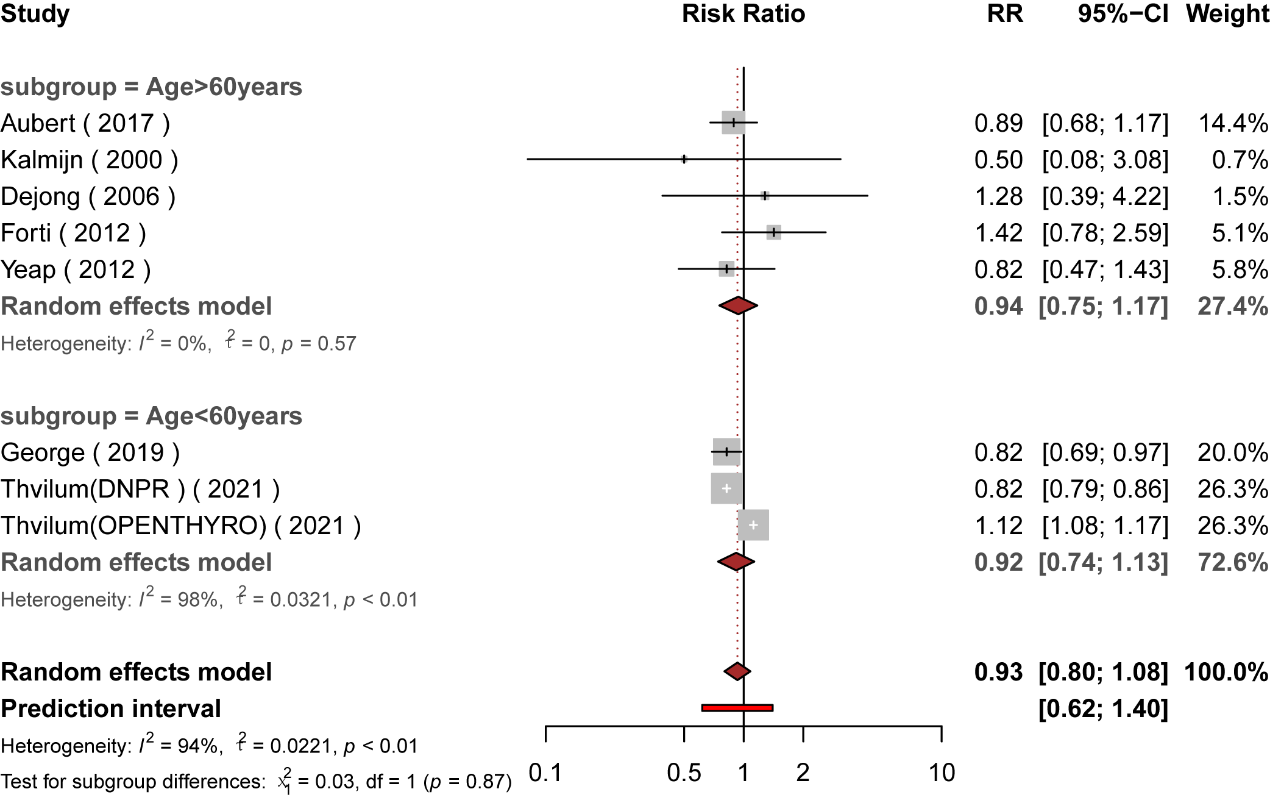


**Adjust for APOEε4**


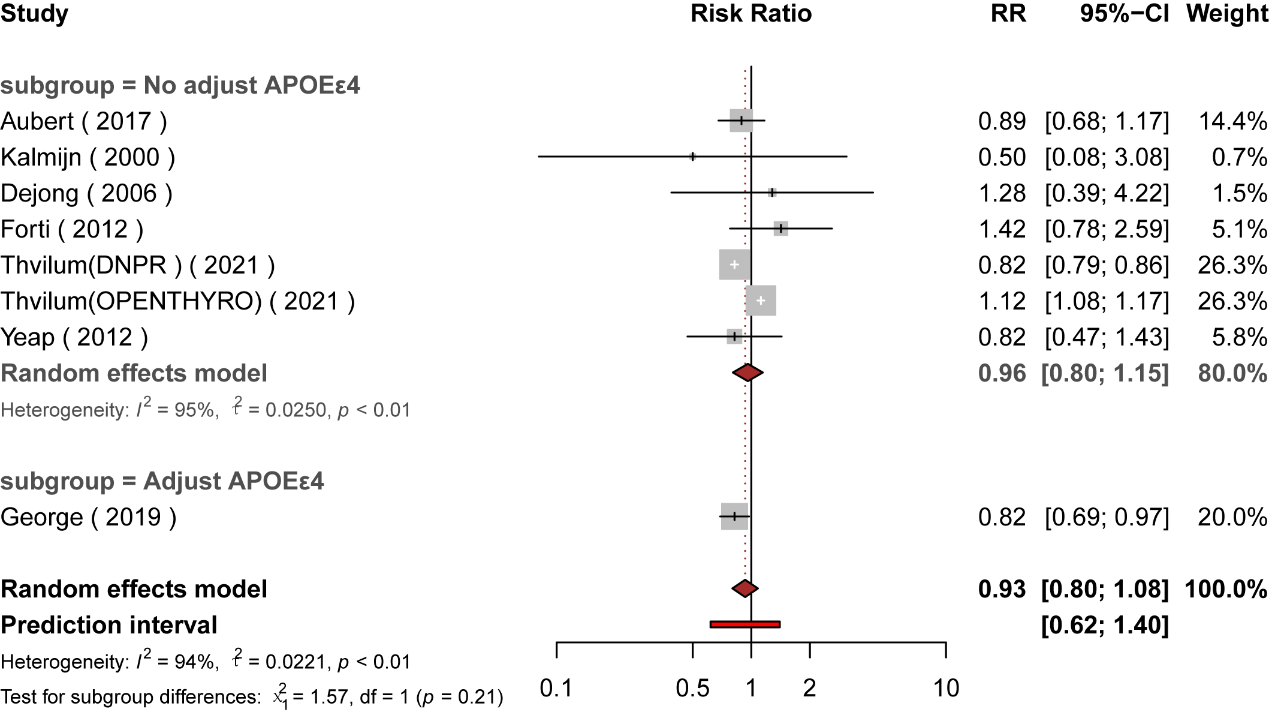


**Follow up**


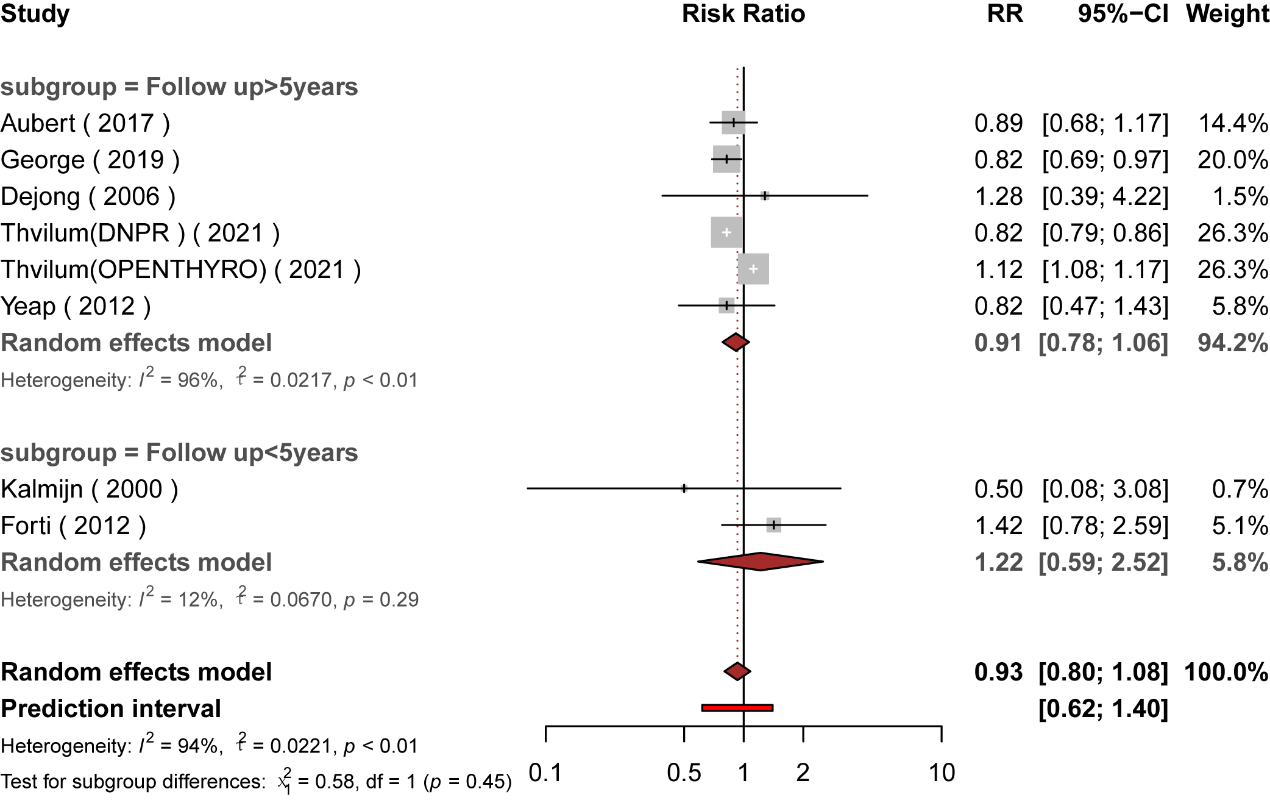


**Source**


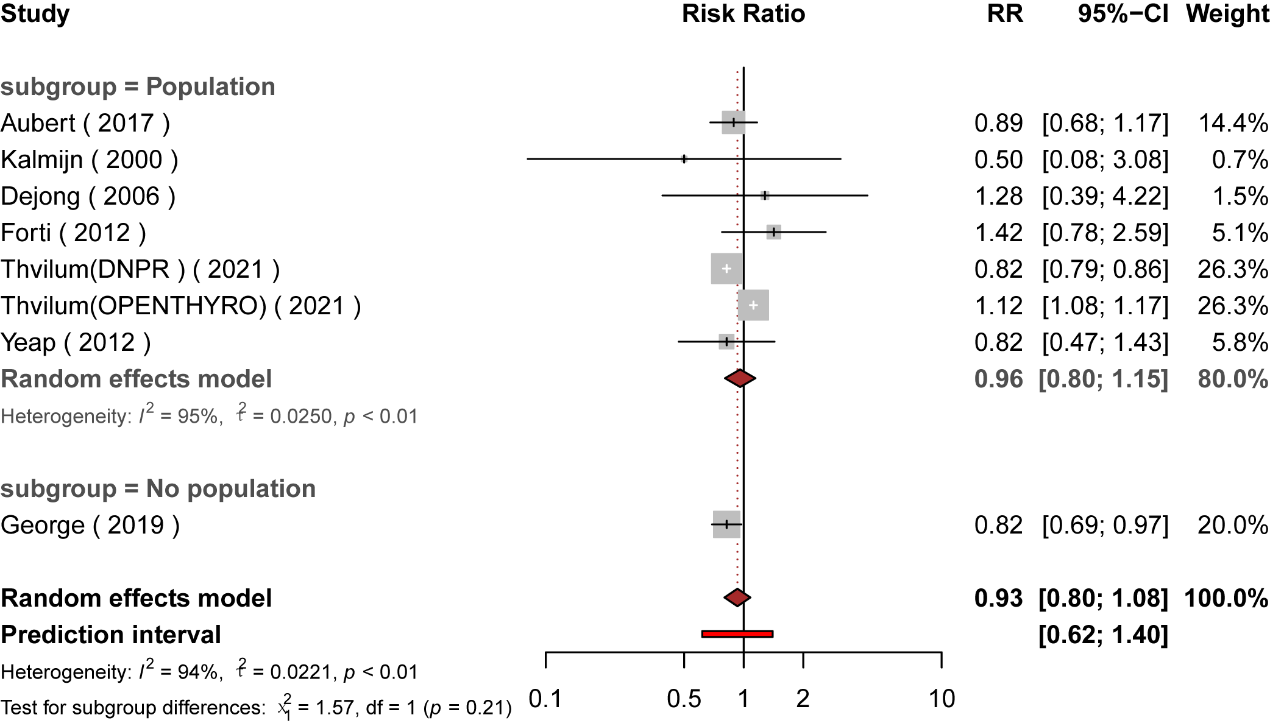


**Region**


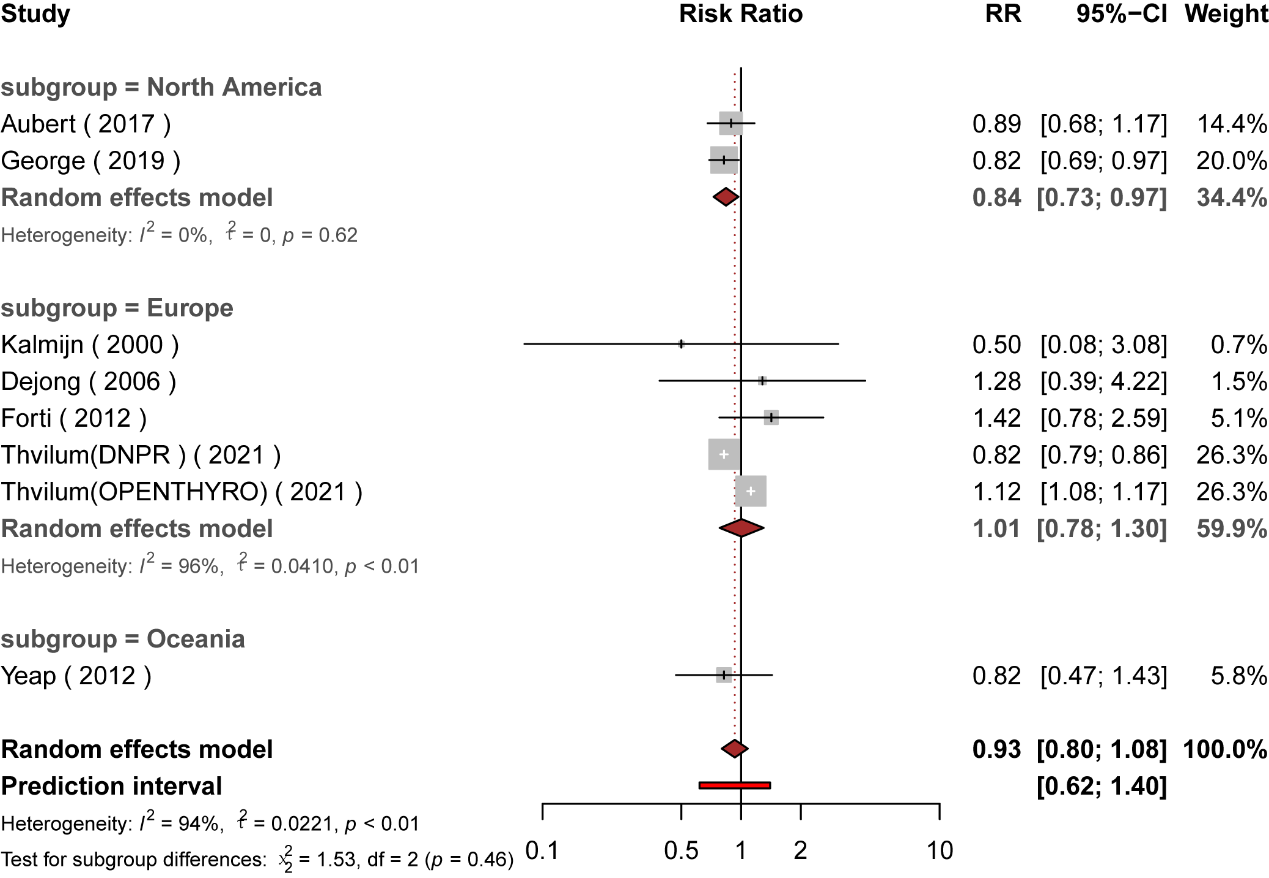


# Supplemental file-12: Funnel plots of hypothyroidism

**Hypothyroidism and dementia**


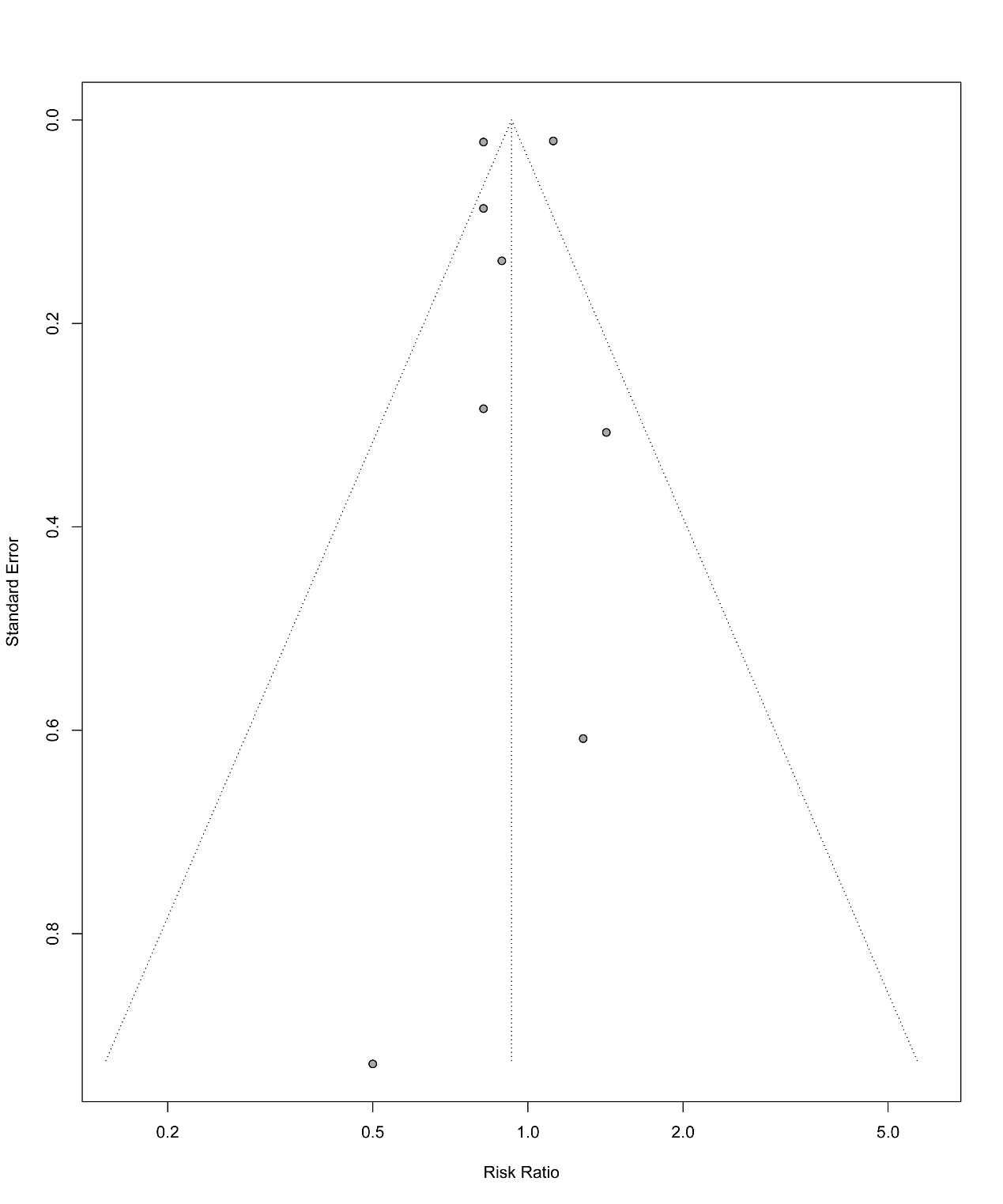


**Hypothyroidism and Alzheimer's Disease**


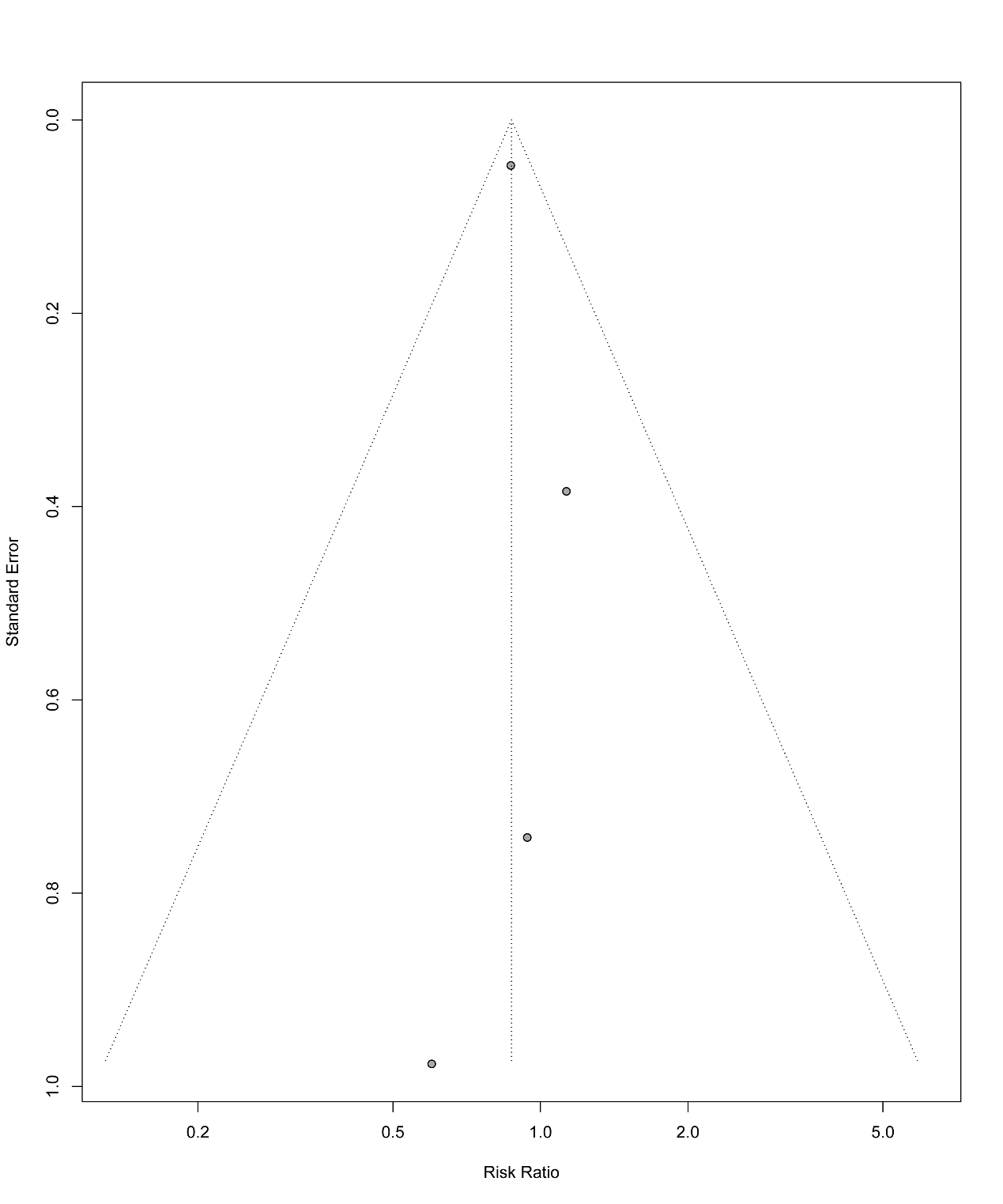


# Supplement file-13: Meta-analysis for subclinical thyroid disease with the combined risk of dementia in cohort studies

**Meta-analysis for** **subclinical hyperthyroidism with the combined risk of dementia in the cohort study**


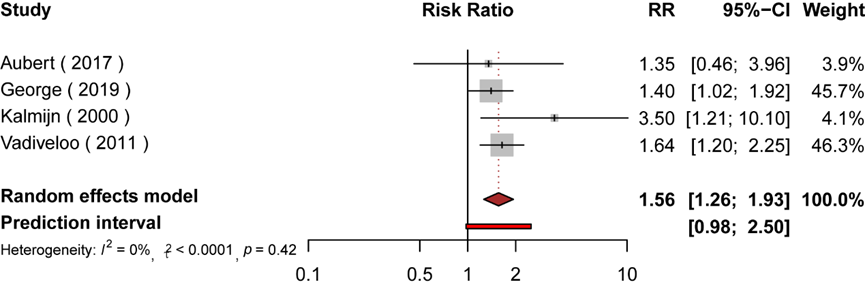


**Meta-analysis for Subclinical hypothyroidism with the combined risk of dementia in the cohort study**

**
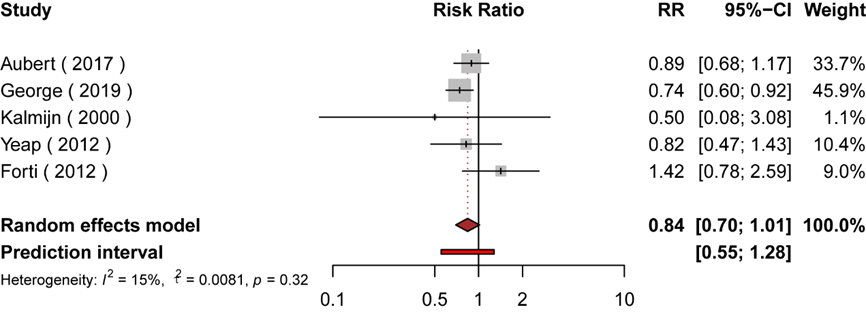
**

# Supplemental file-14: Funnel plots of subclinical thyroid disease

**Subclinical hyperthyroidism and dementia**

**
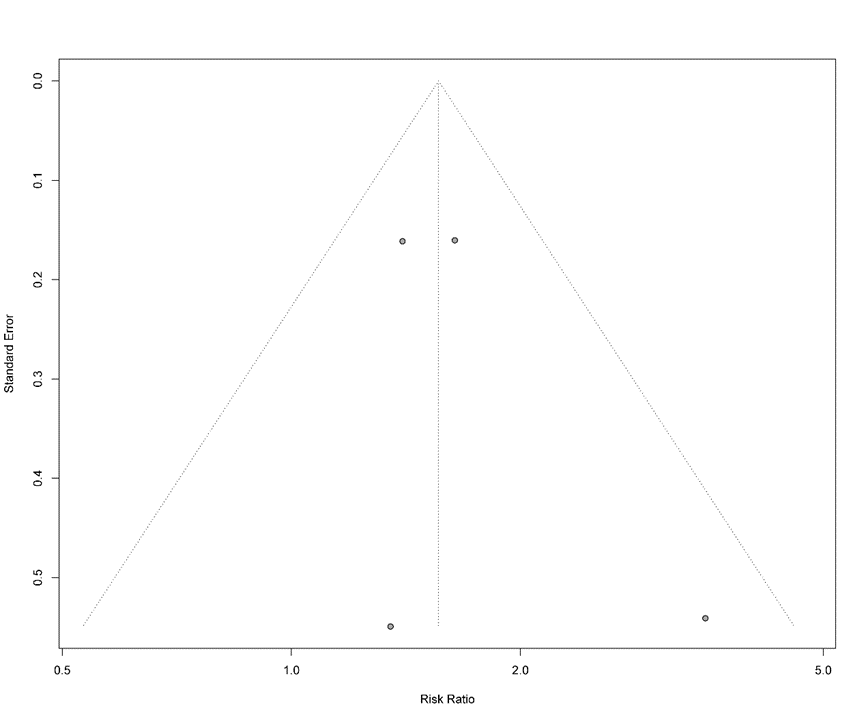
**

**Subclinical hypothyroidism and dementia**

**
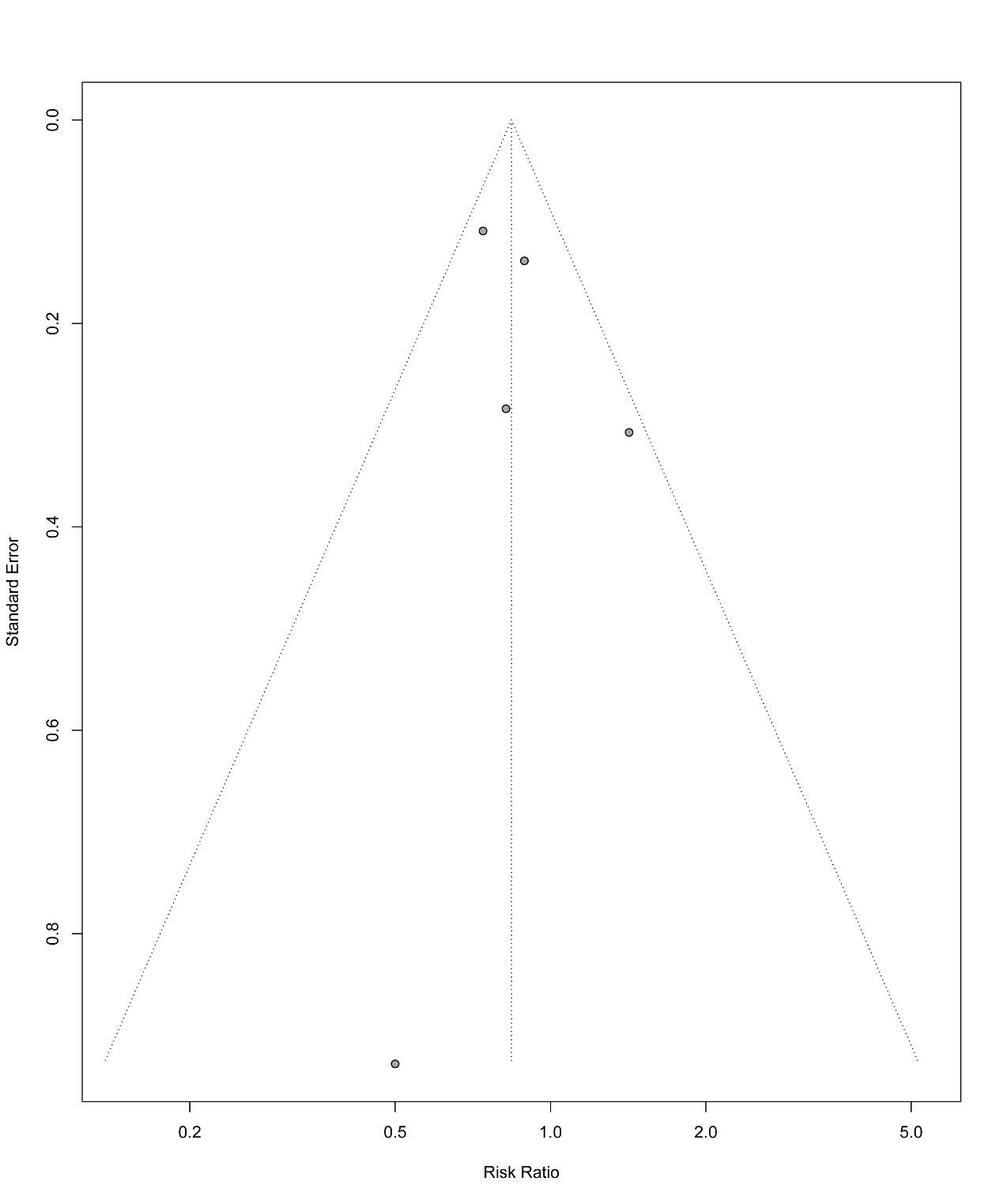
**

# Supplemental file-15: Meta-analysis for hyperthyroidism with the combined risk of dementia in the cross-sectional studies

**
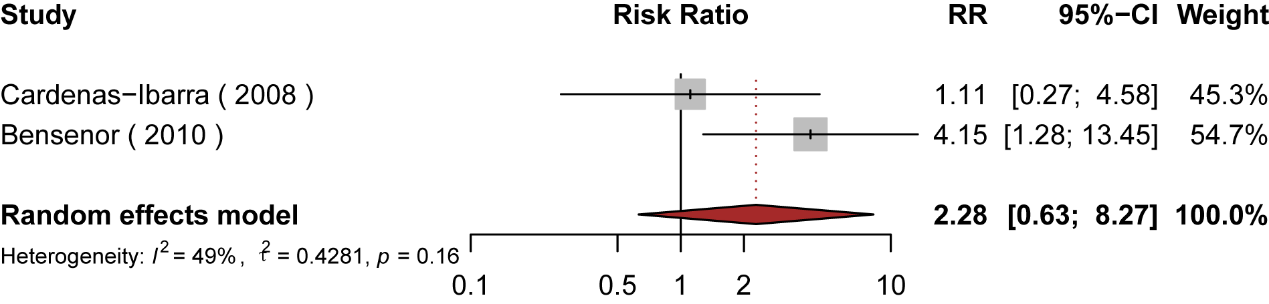
**

# Supplemental file-16: Meta-analysis for hypothyroidism with the combined risk of cognitive impairment and dementia in Cross-sectional studies

**Cognitive impairment**


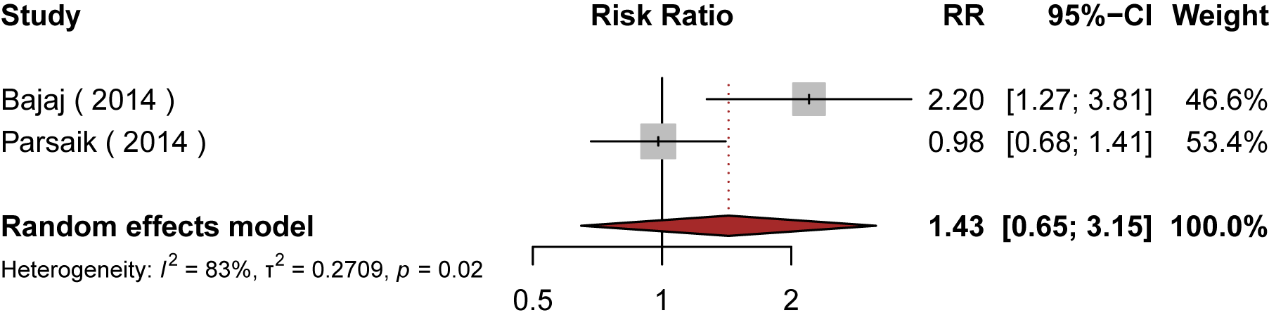


**Dementia**


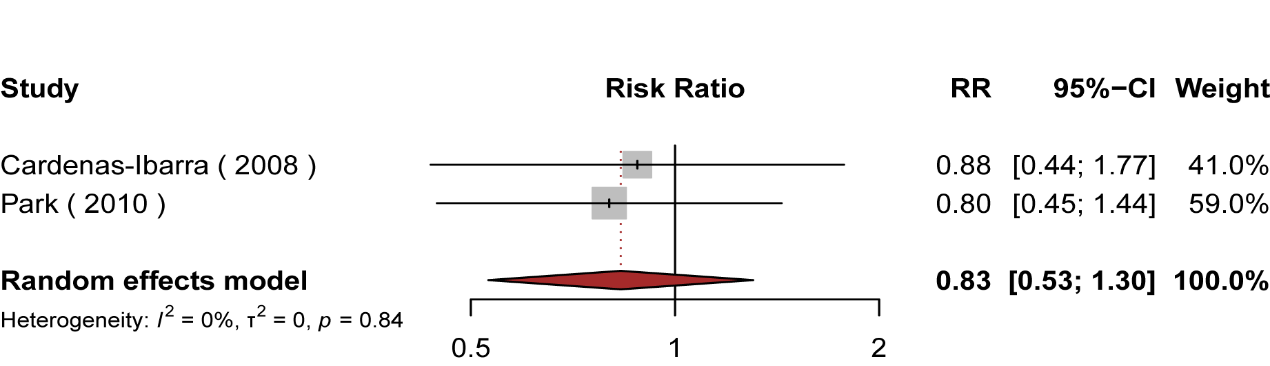

Supplement: Supplementary file 1 [file Data_Sheet_1.docx]
